# Disruption of an Evolutionarily Novel Synaptic Expression Pattern in Autism

**DOI:** 10.1371/journal.pbio.1002558

**Published:** 2016-09-29

**Authors:** Xiling Liu, Dingding Han, Mehmet Somel, Xi Jiang, Haiyang Hu, Patricia Guijarro, Ning Zhang, Amanda Mitchell, Tobias Halene, John J. Ely, Chet C. Sherwood, Patrick R. Hof, Zilong Qiu, Svante Pääbo, Schahram Akbarian, Philipp Khaitovich

**Affiliations:** 1 CAS Key Laboratory of Computational Biology, CAS-MPG Partner Institute for Computational Biology, Shanghai, China; 2 Big Data Decision Institute, Jinan University, Guangzhou, China; 3 Shanghai Key Laboratory of Forensic Medicine, Institute of Forensic Sciences, Ministry of Justice, Shanghai, China; 4 Department of Biological Sciences, Middle East Technical University, Ankara, Turkey; 5 Department of Psychiatry and Friedman Brain Institute, Icahn School of Medicine at Mount Sinai, New York, New York, United States of America; 6 MAEBIOS-TM, Alamogordo, New Mexico, United States of America; 7 Department of Anthropology, The George Washington University, Washington, District of Columbia, United States of America; 8 Fishberg Department of Neuroscience and Friedman Brain Institute, Icahn School of Medicine at Mount Sinai, New York, New York, United States of America; 9 Institute of Neuroscience, Chinese Academy of Sciences, Shanghai, China; 10 Max Planck Institute for Evolutionary Anthropology, Leipzig, Germany; 11 Skolkovo Institute for Science and Technology, Skolkovo, Russia; Massey University, NEW ZEALAND

## Abstract

Cognitive defects in autism spectrum disorder (ASD) include socialization and communication: key behavioral capacities that separate humans from other species. Here, we analyze gene expression in the prefrontal cortex of 63 autism patients and control individuals, as well as 62 chimpanzees and macaques, from natal to adult age. We show that among all aberrant expression changes seen in ASD brains, a single aberrant expression pattern overrepresented in genes involved synaptic-related pathways is enriched in nucleotide variants linked to autism. Furthermore, only this pattern contains an excess of developmental expression features unique to humans, thus resulting in the disruption of human-specific developmental programs in autism. Several members of the early growth response (EGR) transcription factor family can be implicated in regulation of this aberrant developmental change. Our study draws a connection between the genetic risk architecture of autism and molecular features of cortical development unique to humans.

## Abstract 2

A comparative transcriptome study draws a connection between the gene expression alterations in autism and molecular features of cortical development that are unique to humans.

## Author Summary

Autism spectrum disorder (ASD) involves disruptions in cognitive functions related to socialization and communication, which are also among the key behavioral capacities that separate humans from other species. This suggests that ASD may involve alterations in evolutionarily novel, human-specific developmental processes. To test this, we measured developmental gene expression trajectories in the cerebral cortex of autism cases, matched controls, and non-human primates: chimpanzees and macaques. Among a large number of disrupted developmental patterns that we detected in autism, only one, mainly including synaptic genes, was enriched in autism-linked mutations. This suggests that changes in expression of these genes in ASD brains may play a causal role in disease etiology. The same gene set exhibited more developmental expression changes unique to the human brain than any other developmental pattern disrupted in autism, reflecting extension of the synaptic maturation period in humans. Taken together, we show that a recently evolved developmental expression pattern of synaptic genes altered in autism might be associated with intense social learning abilities unique to humans.

## Introduction

Autism spectrum disorder (ASD) is a common neurodevelopmental condition characterized by the disruption of cognitive functions involved in socialization and communication and inclination toward restricted interests and repetitive behaviors [1–4]. The global median prevalence of ASD is estimated at around 0.6% [5], though three to four times higher estimates have also been reported [6,7].

In infants and children, autism can manifest itself within the first 2 y of age as delays or deficits in joint attention, imitation, and communication [4]. Adult ASD individuals can have difficulties in understanding the emotions of others, show low social interaction, display repetitive motor movements, and display different levels of executive dysfunction [4,8]. ASD frequently co-occurs with other neurodevelopmental abnormalities, including language and motor deficiencies [4]. Based on the affected behaviors, a number of central nervous system structures have been implicated to be altered in autism, including the frontal and temporal cortices and the amygdala [4,9].

Imaging studies have shown multiple deviations from average brain development in the brains of ASD individuals. These include increased deficiency in long-range connectivity and low activity in integrative regions [10], which could underlie extremely focused interests in ASD individuals [4]. Another theme is early postnatal brain overgrowth, including excess cortical white and gray matter volumes, that can lead to “megacephalic”-like brain volumes at 2–3 y in boys diagnosed with ASD, which disappear with age due to subsequent brain undergrowth [11]. A similar early overgrowth and later undergrowth pattern was reported for the amygdala [12]. Another observation is aberrant development of white matter fiber tracts in the ASD neocortex, with early acceleration and subsequent deceleration [13]. Stereological work reported >60% higher neuron numbers in ASD children compared to unaffected children [14]. Studies of synapse numbers have also reported an excess of synapse densities; specifically, greater dendritic spine densities were reported for the temporal cortex in ASD brains during postnatal development [15], and in both frontal and temporal cortices in adulthood [16]. The excess of spines in postnatal development appears to increase toward adolescence and has been correlated with reduced developmental spine pruning as a result of high mTOR activity and impaired autophagy [15].

Twin and family studies suggest that cognitive changes observed in autism are more than 50% heritable [17,18]. However, autism has been found to be genetically highly heterogeneous [19,20]. A large number of genetic markers, including segregating and de novo mutations in coding and regulatory regions, copy number variations, and chromosomal mutations, have been linked to ASD by a sum of genetic linkage and genome-wide association studies, while each of the identified genetic variants explains only a minute fraction of disease prevalence, no larger than 1% [4,19,21]. Genes associated with autism in these genetic scans are enriched in two major biological processes: synaptic processes and transcription/chromatin modification [9,21,22]. Some variants are notable for association with other neurodevelopmental conditions, such as schizophrenia, epilepsy, and intellectual disability [21] (which can be concurrent with autism [4]). Interestingly, genes carrying de novo ASD risk mutations were reported to show peak expression in fetal cortical development and in cortical projection neurons in the unaffected brain [23,24]; such genes’ altered activity may thus contribute to abnormal connectivity patterns observed in ASD. Meanwhile, genes carrying segregating (i.e., relatively common) ASD risk alleles were reported to be highly expressed in early postnatal development and have roles in synaptic functions [24], consistent with atypical synaptogenesis in ASD [16].

In addition to genetic analyses, transcriptome comparisons of postmortem neocortex tissue from individuals diagnosed with ASD and from unaffected individuals have revealed a number of phenomena. First, there is massive and consistent downregulation of genes predominantly involved in synaptic functions (despite the spine density excess reported in histological work [15,16]) [25–28]. Second, there is strong upregulation of immune and inflammation-related response genes. Third, synaptic genes, but not immune genes, overlap with putatively causal markers identified in genetic linkage or association studies, indicating that immune upregulation and microglial activation occur downstream of earlier developmental aberrations [25,28] (although immune upregulation and inflammation could also explain important phenomena, such as neuron overgrowth in the ASD brain [28]). Fourth, genes involved in cortical patterning appear dysregulated [25,27], such that expression differences between temporal and frontal cerebral cortices observed in unaffected brains were obscured in ASD brains [25].

One limitation of these transcriptome studies has been their focus on fixed gene expression differences across all ages. Given the transient nature of multiple ASD-related phenotypes [11–14], studying molecular differences between unaffected and ASD brains across postnatal brain development could provide additional insight into ASD etiology. A second limitation pertains to linking the observed molecular changes with the two major behavioral characteristics of ASD-impaired social interaction and restricted behavior. Animal models can help overcome this hindrance [15,29], but here we propose an alternative approach, leveraging upon the notion that the cognitive functions disrupted in autism are among the key behavioral capacities that separate humans from other species [30]. Indeed, the niche that has allowed ratchet-like cultural accumulation in the human lineage was founded upon the evolution of a powerful set of social skills, including theory of mind, imitation, empathy, and cooperation [31–35]. This suggests that some ASD phenotypes may represent disruption of evolutionarily novel, human-specific developmental processes. Accordingly, developmental shift in expression of synaptic genes in the frontal cortex has been shown to be one of the most prominent human-specific changes in postnatal brain development [36,37].

In this study, we investigate the developmental mechanisms underlying ASD, employing transcriptome-wide analysis of expression variation throughout postnatal cortical development, starting from the earliest ages at which autism can be diagnosed. We report a group of co-expressed genes involved in synaptic function and development, which show divergent expression between ASD and unaffected brains from childhood onward. We further show this gene set’s connection to evolutionarily novel developmental features characteristic of the human neocortex.

## Results

### Gene Expression in Autistic and Control PFC Development

**Fig 1:**
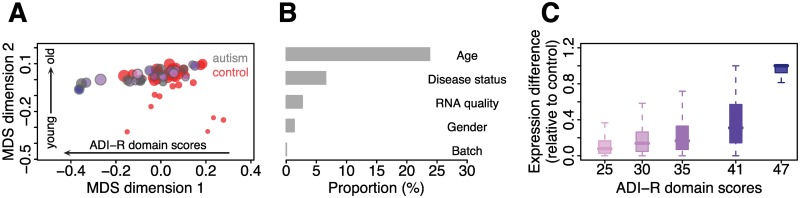
Gene expression in PFC development in autism and control groups. (A) Transcriptional similarity among autism and control cases during PFC development, visualized using multidimensional scaling (MDS) with expression correlation as distance (S2 Data). Each circle represents an individual. The size of the circles is proportional to the individuals’ age (smaller circles correspond to younger individuals). The border colors represent different groups (black: autism cases, red: controls). The filled colors with different shades indicate the levels of cognitive decline for autistic individuals (darker purple correspond to more severe cases). The first dimension correlates with levels of cognitive decline (Pearson correlation, *r* = 0.42, *p* < 0.001) and the second with age (Pearson correlation, *r* = 0.73, *p* < 0.001). (B) Proportions of the total expression variation explained by disease status, age, sex, RNA quality, and sample batch across all 12,557 genes expressed in autism and control cases. (C) Distance between the expression trajectory of control group and autism cases with different levels of Autism Diagnostic Interview-Revised (ADI-R) scores. Darker shade of purple corresponds to higher ADI-R scores.

To determine the developmental dynamics of gene expression changes in autism, we measured transcript levels in the prefrontal cortex (PFC) of 34 autism cases (2–60 y old) and 40 controls (0–62 y old) using RNA sequencing (RNA-seq; Fig 1A and S1 Table). Based on more than 700 million sequence reads, we quantified the expression of 12,557 protein-coding genes in 63 individuals (S1 and S2 Tables and S1 Data). The age of the individuals explained 23.8%, and autism diagnosis 6.6%, of the of the total expression variation. By contrast, other factors, such as gender and RNA quality and sample preparation batch as confounds, collectively explained 4.1% (Fig 1B).

A total of 1,775 genes were differently expressed between autism cases and unaffected controls in our data (ANCOVA, *p* < 0.01, permutation-based false discovery rate (FDR) = 0.078). These genes overlapped significantly with expression differences determined using an alternative statistical procedure, the likelihood ratio test implemented in the DESeq2 package [30], set to the same FDR level (Fisher's exact test, *p* < 0.0001, odds ratio = 38.7), as well as differentially expressed genes reported in the autistic frontal cortex via microarray-based study [25] (Fisher's exact test, *p* < 0.0001, odds ratio = 2.46) and with genes identified by reanalysis of published microarray data [25,36] (Fisher’s exact test, *p* < 0.0001, odds ratio = 1.54). Furthermore, the direction of expression change in autism determined in our study was concordant with results obtained using microarrays [25,36], even though 50 of the 63 individuals did not overlap between studies (Pearson correlation, *r* = 0.68, *p* < 0.0001; S1 Fig).

**Fig 2:**
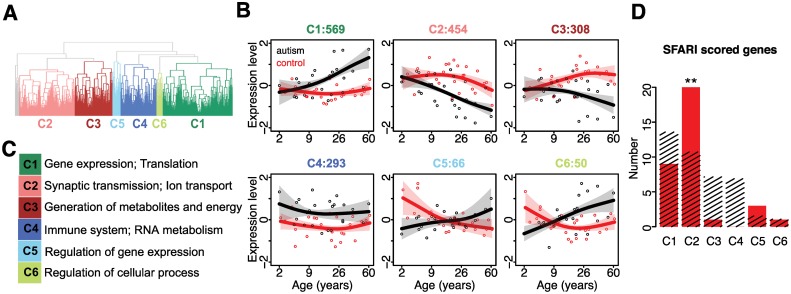
Developmental dynamics of gene expression changes in autistic brains. (A) Hierarchical clustering of 1,775 genes differently expressed between autism cases and unaffected controls (S3 Data). (B) Expression patterns of the six major gene clusters. The *x*-axis shows the age information on the (age)^1/4^ scale; the *y*-axis shows the expression levels standardized to mean = 0 and standard deviation = 1 before plotting. The points represent mean expression levels in each individual (red: controls; black: autism cases); the lines show cubic spline curves fitted to the individual data; the shaded areas show the standard deviation of the spline curves within a cluster. The cluster number and the number of genes within the cluster are shown on top of the panels. (C) Summary of functional pathways enriched in each cluster (S3 Table). (D) Overlap between genes with 1–4 scores in SFARI AutDB linked to autism by genetic association studies and six major clusters of expression changes in autism (S4 Data). The red bars show the numbers of overlapping genes; the streaked bars show the mean numbers of overlapping genes expected by chance, estimated by 1,000 permutations of cluster labels. The symbols above the bars show the significance based on 1,000 permutations of cluster labels (**: *p* < 0.01).

Unsupervised clustering of the 1,775 genes resulted in six major patterns of developmental expression profiles separating autism and control groups (Fig 2A and 2B; S2 Fig). In agreement with previous observations [25–27], transcripts upregulated in autism (cluster 4) were preferentially involved in immune functions, while transcripts downregulated in autism (cluster 2) were involved in neural functions, including calcium signaling and long-term potentiation pathways, as well as Gene Ontology (GO) terms associated with synaptic transmission and cell–cell signaling (one-sided hypergeometric test, corrected *p* < 0.05; Fig 2C and S3 Table). In addition to these previously reported functions, we found expression changes enriched in cellular metabolic and biosynthetic processes (cluster 1), generation of precursor metabolites and energy and catabolic process (cluster 3), regulation of transcription (cluster 5), and regulation of cellular process (cluster 6). Clustering based on log2-transformed expression values (S3 and S4 Figs), as well as expression values of all detected genes, demonstrated robustness of clustering results, especially for cluster 2 genes (S5 and S6 Figs). Cluster 2 genes also overlapped significantly with genes reported by other studies of transcriptome changes in autism [25,28] (Fisher's exact test, *p* = 0.0043 and odds ratio = 1.68 for genes in [28]; *p* = 0.0004 and odds ratio = 2.89 for genes in [25]). Notably, the extent of the expression difference between autism and control cases positively correlated with the Autism Diagnostic Interview-Revised (ADI-R) scores (Pearson correlation, *r* > 0.5, *p* < 0.05; Fig 1C and S4 Table). Patterns of age-related expression divergence were not, however, driven by these extreme cases, as re-plotting clusters based only on autism samples with moderate ADI-R scores confirmed all cluster profiles (S7 Fig).

### Frequency of Autism-Associated Genetic Variants in Gene Expression Clusters

To test whether any of the detected expression changes might be linked with the cause of disease, we assessed the occurrence of genes with DNA sequence polymorphisms and mutations associated with ASD in each of the six clusters. Notably, genes in cluster 2, but not in the other five clusters, contained a significant excess of genes with genetic variants associated with ASD, based on analysis of all genes extracted from SFARI AutDB (791 genes) [38], category 1–4 genes from SFARI AutDB (375 genes), genes from AutismKB database (977 genes) [39], as well as genes with de novo mutations associated with autism collected from four exome sequencing studies (permutation test, *p* < 0.05 for all tests; Fig 2D and S8 Fig) [8,10–12]. This result was not affected by gene length differences between clusters (S9 and S10 Figs). This suggests that disruption of the synaptic gene developmental expression pattern, represented by cluster 2, may represent one of the primary causes of ASD, while other expression changes represent its consequence.

### Localization of Genes Affected in Autism in Cell Types and Cortical Layers

To further investigate the functional roles of genes showing significant expression changes in autistic brains, we assessed the distribution of genes in each of the six clusters among brain cell types and cortical layers. Based on mouse brain cell type makers reported in [16], we found that cluster 2 were significantly over-represented among genes highly expressed in three types of neurons assessed in [16], CA1 pyramidal cells, interneurons, and S1 pyramidal cells, but not in non-neuronal cells. Cluster 5 genes were over-represented among S1 pyramidal cell markers, and cluster 3 genes tended to overlap with interneuron markers (S11 Fig). These observations were generally consistent with results based on mouse neuronal marker genes reported in [40] (S12 Fig).

Given enrichment of cluster 2, 3, and 5 genes in neuronal markers, as well as enrichment of cluster 2 genes in GO terms “synaptic transmission” and “cell–cell signaling,” we further collected genes annotated to be predominantly localized in the presynapse, postsynapse, synaptic vesicle, axon, dendrite, and remaining neuron locations based on GO. Genes in cluster 2 were significantly over-represented among genes located in synaptic vesicles and dendrites, but not in other neuron locations (S13 Fig). By contrast, genes in the other clusters showed no significant over-representation in any of the subcellular structures of neurons. Taken together, these observations further support the notion that cluster 2 genes are linked to synaptic transmission and neuronal signaling.

Using marker genes of cortical layers 2–6 identified in rhesus macaque brains [14], we further found that genes in cluster 4 show significant over-representation among cortical layer 5 markers (S14 Fig). This observation agrees with the notion implicating genes expressed in deep cortical layers during mid-fetal embryonic development as pertinent to ASD [35]. Intriguingly, genes in clusters 2 and, to a lesser extent, cluster 3 were significantly over-represented among cortical layer 2 markers, which is also in line with observations of gene expression dysregulation in adult ASD patients in cortical layers 2/3 (S14 Fig) [24].

### Comparison of Autistic and Human-Specific Developmental Expression Profiles

**Fig 3:**
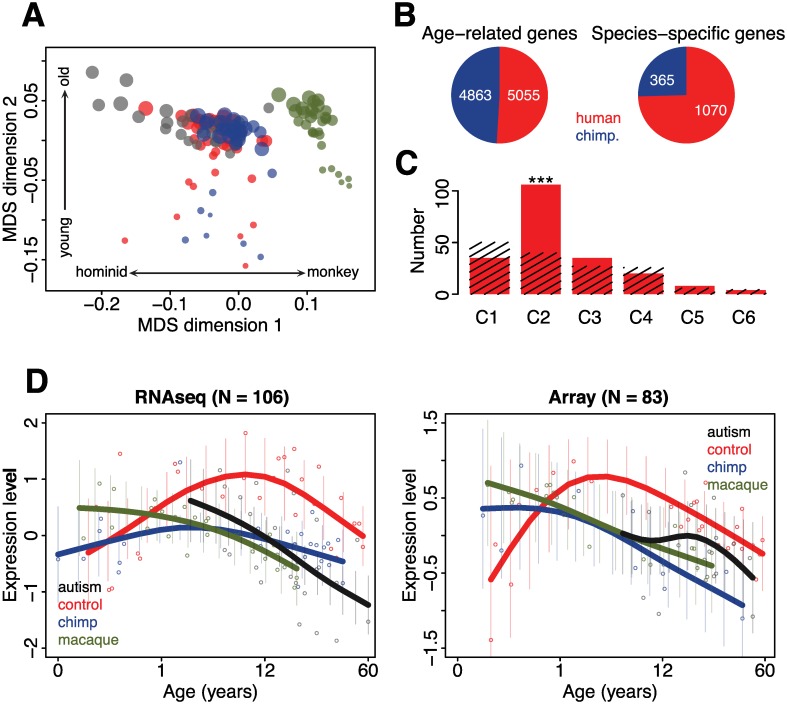
Relationship between human-specific and autism-related developmental changes. (A) A global view of transcriptional similarity among autism and control cases, as well as chimpanzees and macaques, visualized using MDS with expression correlation as distance. Each circle represents an individual. The size of the circles is proportional to the individuals’ age (smaller circles correspond to younger individuals). The colors represent different species (black: autism cases; red: controls; blue: chimpanzees; green: macaques). The first dimension correlates with hominid–monkey divergence (Pearson correlation, *r* = 0.80, *p* < 0.001) and the second with age (Pearson correlation, *r* = 0.81, *p* < 0.001). (B) Numbers of genes showing age-related and species-specific developmental profiles (red: human controls; blue: chimpanzees). (C) Overlap between genes showing human-specific developmental profiles and six major clusters of expression changes in autism (S4 Data). The red bars show the numbers of overlapping genes; the streaked bars show the mean numbers of overlapping genes expected by chance, estimated by 1,000 permutations of cluster labels. The symbols above the bars show the significance of the overlap based on 1,000 permutations (***: *p* < 0.001). (D) Expression profiles of genes showing human-specific developmental profiles and expression divergence between autism and control cases in cluster 2 measured by RNA-seq (left panel) or microarrays (right panel). The *x*-axis shows the age information on the (age)^1/4^ scale; the *y*-axis shows the expression levels standardized to mean = 0 and standard deviation = 1 before plotting. The points represent mean expression levels in each individual (red: controls; black: autism cases; blue: chimpanzees; green: macaques), the lines show cubic spline curves fitted to the individual data, and the error bars show standard deviation of the spline curves. The numbers of genes used for plotting are shown on top of the panels.

ASD affects cognitive functions involved in socialization and communication [1–3], functions that have also been suggested to underlie main aspects of human cognitive uniqueness relative to other primates [30–33]. To test whether genes whose ontogenetic expression patterns are changed in autism display human-specific properties, we measured gene expression levels in the PFC of 39 chimpanzees (0–43 y old) and 36 rhesus monkeys (0–21 y old), using the same RNA-seq procedure as for the human samples (Fig 3A, S1 and S2 Tables). Of the 12,557 genes detected in the 38 human, 31 chimpanzee, and 31 macaque samples with good RNA quality (S1 Table), 1,070 (8.5%) showed developmental expression profiles specific to humans (ANCOVA test, *p* < 0.01, permutation-based FDR < 0.01). The expression of these genes across PFC development correlated well with published human, chimpanzee, and rhesus monkey data measured using microarrays (mean *r* = 0.7, *p* < 0.0001; S15 Fig) [36,37]. Furthermore, in agreement with previous observations [36,37], the proportion of developmental gene expression profiles specific to humans exceeded the proportion of chimpanzee-specific profiles by approximately three-fold (Fig 3B).

Remarkably, among six clusters of genes differentially expressed between autism and control groups, only cluster 2 contained a substantial and significant excess of genes showing human-specific expression profiles during PFC development detected in either RNA-seq or microarray datasets (permutation test, *p* < 0.001; Fig 3C), with 82% of them converging on the same common human-specific developmental expression pattern in both datasets (Fig 3D). Among these genes, the functional categories synaptic transmission, calcium signaling pathways, cell communication, as well as 14 other pathways associated with synaptic functions are highly overrepresented (one-sided hypergeometric test, corrected *p* < 0.05; S5 Table). Although based on a small number of samples spanning the critical age interval, previous results [36,41–43], as well as the current data, indicate that peak expression of synaptic genes is substantially delayed and extended in human PFC development compared to both chimpanzees and macaques: from 0–2 years in non-human primates to 4–10 years in humans (Fig 2D and S16 Fig). In the autistic brain, expression of synaptic genes starts decreasing from the earliest measured time point, 2 years of age, much earlier than in the control individuals (Fig 2D). This might imply that the timing of expression of synaptic genes in PFC is accelerated in autism, which resonates with premature PFC development described for ASD [11,44]. Furthermore, the average profile of all synapse-related genes follows the expression trajectory closely resembling the trajectory of cluster 2 genes with human-specific expression (S17 Fig). This observation further implies an accelerated dynamic of synaptic maturation during the first years of postnatal development in autism, suggesting a potential mechanism for the resulting disruption of cognitive functions.

### Regulation of Aberrant Expression of Synaptic Genes in Autism

To assess which regulatory mechanisms might drive the aberrant expression of cluster 2 genes in autism, we first examined reported changes in the density of trimethylated histone H3K4 (H3K4me3, an epigenetic mark that on a genome-wide scale broadly correlates with active promoters) in neurons isolated from PFC of autism and control cases of different ages [45]. We found a significant excess of positive correlations between gene expression and H3K4me3 density changes in autism for genes in clusters 3 and 5 (one-sided Wilcoxon test, *p* < 0.05; S18 Fig). Notably, clusters 3 and 5 contain genes preferentially expressed in neurons (S11 and S12 Figs). Thus, H3K4me3 density changes might be associated with gene expression dysregulation in neuronal cells in the autistic brain and are not specific to the aberrant regulation of synaptic maturation.

**Fig 4:**
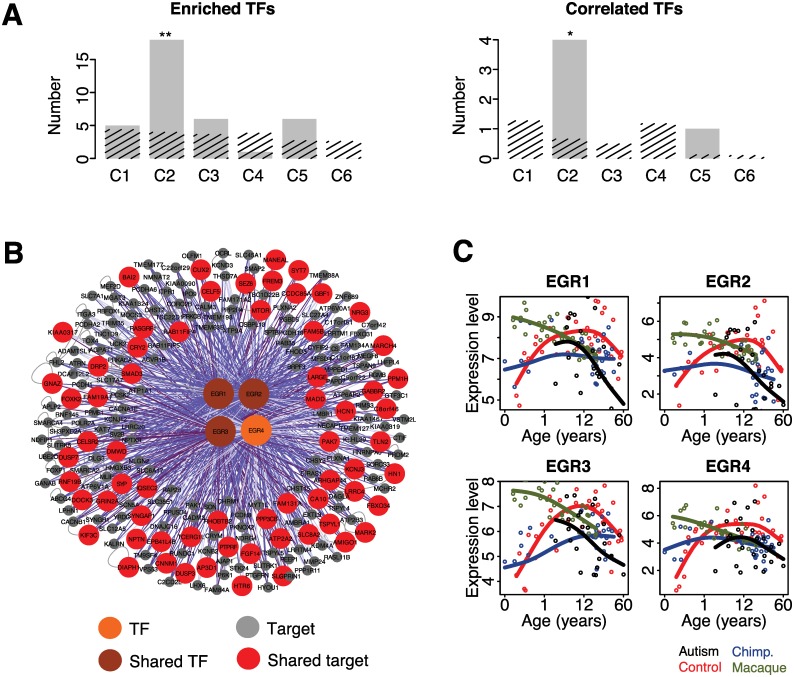
Potential regulators of gene expression changes in autism. (A) The numbers of TFs with significant binding site enrichment in genes’ promoter regions in the six major clusters of expression changes in autism (left; S4 Data). The numbers of TFs showing binding site enrichment, as well as significant positive correlation with expression profiles of its predicted target genes, within a cluster (right; S4 Data and S5 Data). The gray bars show the observed TF numbers, and the streaked bars show the mean number of TFs expected by chance, estimated by 1,000 permutations of cluster labels. The symbols above the bars show the significance based on 1,000 permutations of cluster labels (**: *p* < 0.001; *: *p* < 0.1). (B) Predicted regulatory network driving expression changes in autism represented by cluster 2 profile. The large circles represent regulatory TFs (orange). The darker shade of colors represents regulators associated with human-specific extension of cortical synaptogenesis [36,37]. The smaller circles represent cluster 2 targets of displayed TFs showing evolutionary conserved (gray) and human-specific expression (red) during PFC development. The purple lines indicate predicted TF-target gene interactions (dark purple: correlation coefficient more than 0.6; purple: correlation coefficient less than 0.6 but more than 0.3; light purple: correlation coefficient less than 0.3), and the gray lines indicate protein–protein interactions collected from iRefindex database [6]. (C) Expression profile of the *EGR1-4*. In each panel, points indicate individuals (red: controls; black: autism cases; blue: chimpanzees; green: macaques); lines show cubic spline curves.

We next tested the roles of transcription factors (TFs) in the regulation of expression changes found in autism using target site predictions from Transfac [46]. Interestingly, we found a significant enrichment of binding sites for 18 TFs in the promoters of cluster 2 genes, but not genes in the other five clusters (permutation test, *p* < 0.01 for TFs; Fig 4A). Furthermore, among TFs showing significant binding site enrichment in cluster 2 genes, the expression patterns of four of these, early growth response proteins 1–4 (EGR1–4), were correlated with the expression of their predicted targets (BH corrected Mann-Whitney U [MWU] test, *p* < 0.05; Fig 4A). The authenticity of the EGR target predictions used in this analysis was further confirmed using data from chromatin immunoprecipitation followed by sequencing (ChIP-seq) experiments conducted using EGR1 and EGR2 antibodies by the ENCODE project [22] and by other studies [9,16] collected in a total of seven cell lines (S6 Table and S19 Fig).

The four TFs are early growth response proteins (EGR1–4), shown to be involved in the regulation of neuronal function, including synaptic activity [47], neuronal plasticity [48], and neuronal cell death (Fig 4B and 4C) [49]. Strikingly, three of the four *EGRs* (*EGR1-3*) have been previously identified as potential regulators of the human-specific delay in the timing of cortical synaptogenesis [36], while less than one would be expected by chance (permutation test, *p* < 0.001). Furthermore, genomic regions surrounding *EGR1* display an excess of genetic mutations linked to ASD (permutation test, *p* < 0.05 for *EGR1*; S20 Fig) [50]. Together, these observations support the conclusion that *EGRs* have a role in the aberrant regulation of synaptic maturation in autism.

## Discussion

Though multiple studies have considered aberrant gene expression changes in ASD, associating these with behavioral phenotypes has continued to be a challenge. The evidence to date has pointed towards a causal role for down-regulation of synapse-related genes [25–27]. Our results, based on time-series analysis of postnatal brain development in ASD, provide further insight into this observation. First, among all expression changes detected in autism, we find that only one major cluster is functionally linked to cognition-related physiological processes: cluster 2 genes, but not genes in the other clusters, show significant localization preference towards synaptic vesicles and dendrites of neurons and are significantly enriched among genes annotated to be involved in synaptic transmission (Fig 2C). Second, we demonstrate that genes in cluster 2, albeit representing less than a quarter of genes with expression changes in autism, are enriched in genetic mutations linked to the disease (Fig 2D). Third, our data shows that down-regulation of synaptic genes in autism is already present by early childhood and likely arises from altered synaptic development dynamics at earlier postnatal phases in ASD (Figs 2B and 3D).

Thus, even though autism clearly involves a highly heterogeneous set of etiologies and genetic conditions, aberrant developmental expression of synaptic genes might represent one of its causes. This notion largely concurs with observations of greater neuron numbers, pruning deficits, and rapid brain growth in children diagnosed with ASD [14–16,51–53] and has the potential to provide a mechanistic basis for the short-range synaptic hyper-connectivity and long-range hypo-connectivity characteristic of the neocortex observed in ASD individuals [10,54–57]. How atypical synaptic gene expression influences these diverse characteristics, how much atypical expression is influenced by atypical environmental input, and whether synaptogenesis in different brain regions are similarly affected, remain to be resolved.

Fourth, we find indication that the EGR family of zinc-finger transcription factors functions as regulators of atypical synaptic expression in ASD, identified in cluster 2. A number of observations support the role of EGRs in ASD etiology: (a) as reported earlier, EGR1 displays an excess of de novo mutations (S20 Fig) [50]; (b) variants disrupting EGR binding sites in the Contactin-associated protein-like 2 (*CNTNAP2*) promoter were reported as risk factors for ASD [58]; (c) MECP2, in which mutations cause Rett syndrome [29,59], was found to be regulated by EGR2 in a positive feedback loop [60]; (d) compared to controls, *EGR2* expression is significantly lower in Rett syndrome and ASD brain samples [60], as well as in ASD lymphoblastoid cells [61]. Multiple studies indicate the role of EGR family TFs in learning and synaptic function [47,62], and recent work showed that EGR1 directly regulates GABA receptor subunits in the hippocampus [63]. It is therefore tempting to propose that the excitatory-inhibitory imbalance, suggested to underlie ASD, could be directly EGR-driven.

Finally, our measurements taken at different points of cortical development allow us to compare the transcriptome dynamics of brain development in ASD with evolutionary differences between humans and other primates. Comparative psychology has made two distinct observations regarding ASD cognition and human evolution: (a) impaired social cognition, which could be evolutionarily deleterious in hominin societies [30], and (b) hyper-systemizing and attention to detail, a skill that could be beneficial and therefore positively selected [64]. In our data, the dynamics of synaptic expression changes seen in autism in the PFC represent a disruption of one of the most prominent human-specific developmental changes detected in neurotypical brains: delayed expression of synaptic genes and extended period of synaptic maturation [36], a trait thought to be critical for human social and linguistic learning [44]. This observation might explain the impairment of cognitive aspects suggested to be uniquely human [30] in ASD.

## Materials and Methods

### Sample Collection

The study was reviewed and approved by the Institutional Animal Care and Use ethics committee at the Shanghai Institute for Biological Sciences, CAS. Informed consent for use of human tissues for research was obtained in writing from all donors or their next of kin. All non-human primates used in this study suffered sudden deaths for reasons other than their participation in this study and without any relation to the tissue used. We used prefrontal cortex (PFC) samples from postmortem brains of 40 cognitively unaffected human controls (0–62 years old), 34 autism cases (2–60 years old), 39 chimpanzees (0–43 years old), and 36 rhesus monkeys (0–21 years old) (S1 Table). Unaffected human samples were obtained from the NICHD Brain and Tissue Bank for Developmental Disorders at the University of Maryland, USA, and the Maryland Brain Collection Center, Maryland, USA. Autism samples were obtained from NICHD Brain and Tissue Bank for Developmental Disorders and the Harvard Brain Tissue Resource Center. Chimpanzee samples were obtained from the National Chimpanzee Brain Resource (NS092988), the Alamogordo Primate Facility, New Mexico, USA, the Anthropological Institute and Museum of the University of Zürich-Irchel, Switzerland, and the Biomedical Primate Research Centre, the Netherlands. Rhesus monkey samples were obtained from the Suzhou Experimental Animal Center, China. PFC dissections were made from the frontal part of the superior frontal gyrus and all samples contained an approximately 2:1 grey matter to white matter volume ratio.

### RNA Sequencing (RNA-seq)

Total RNA was isolated using Trizol reagent (Invitrogen, Carlsbad, California). Sequencing libraries were prepared with TruSeq RNA Sample Preparation Kit (Illumina) according to manufacturer’s instruction. Briefly, poly-T oligo-attached magnetic beads were used to isolate long polyadenylated RNA from 1 μg of total RNA. After fragmentation, first-strand cDNA was reverse transcribed with random hexamer-primers, followed by second-strand cDNA synthesis, end repair, adenylation of 3′ ends, and ligation of the adapters. Fragments were then enriched by PCR and sequenced on the Illumina Hi-seq 2000 system in seven lanes of one flow cell, using the 100-bp single-end sequencing protocol. All samples were randomized prior to library preparation and RNA sequencing.

### RNA-seq Data Processing

In total, we obtained 1,495,421,886 RNA-seq reads, with an average sample coverage of approximately 10 million reads (S2 Table). The procedures for data processing were previously described [36]. Briefly, the raw sequencing reads were mapped to each specie’s reference genome (hg19, panTro3, and rheMac2) and the splice junctions using Bowtie [65]. The splice junction annotation was constructed using UCSC LiftOver tool (http://www.genome.ucsc.edu/cgi-bin/hgLiftOver) by combining annotated conserved exons within a gene. Allowing up to three mismatches, 86% of the reads could be mapped to the corresponding genome, 92% of them uniquely (S2 Table). Only uniquely mapped reads were used in the downstream analysis. Human gene annotation was downloaded from Ensembl (v66; http://www.ensembl.org). Chimpanzee and rhesus monkey gene annotations were constructed from human gene annotation using the LiftOver tool. Gene annotations were filtered to exclude transcripts with large size differences among the three species; the difference in transcript length between species should be smaller than the length of the shortest transcript. Gene expression levels of transcripts were measured as RPKM values (reads per kilobase per million; S1 Data). If a gene contained multiple transcripts, the expression level of the longest transcript was chosen. The expression levels were standardized to mean = 0 and standard deviation = 1 before plotting. It should be noted that the expression profiles of autism and control samples were normalized together.

### Identifying the Differentially Expressed Genes in Autism

We identified differential expression between autistic samples and unaffected controls using analysis of covariance (ANCOVA) [66] as described in [36,37,67,68]. The detailed description of the test applied is provided in the S1 Text. Briefly, for each gene, we chose the best polynomial regression model with age as predictor and RPKM values as response, using a family of polynomial regression models and the “adjusted r2” criterion. ANCOVA was then used to test whether the regression model with disease-status parameters was significantly better than the model with common parameters for both disease and control groups. The F-test was used to compare the null model (with no disease-status parameters) with the alternative model (with disease-status parameters). The permutations were performed by dividing samples into eight age intervals and permuting disease-status identifiers within each interval in order to preserve the age structure of the data. The FDR was calculated by 1,000 random permutations of sample labels. One thousand, seven hundred seventy-five genes under F-test *p* < 0.01 and FDR = 0.078 were selected as differentially expressed genes in autism.

To test the robustness of the differential expression in autism calculated by ANCOVA, the likelihood ratio test in the nbinomLRT function in R package “DESeq2” [30], with counts of reads per gene as inputs, was used to test the significance of changes in deviance between a full mode (~ age + group) and reduced mode (~age), where age is in days and group is the disease status. *P*-values in the likelihood ratio test were adjusted by Benjamini-Hochberg (BH) adjustment. One thousand, seven hundred thirty-two genes were identified with adjusted *p*-values less than 0.078, under the same cutoff in ANCOVA analysis.

To further evaluate the differential expression in autism, we estimated its overlap with the differentially expressed genes in autistic brains reported previously [25], as well as the differentially expressed genes, by reanalyzing the published microarray data by ANCOVA, in which we re-normalized the expression profiles from a primates microarray dataset and an autism microarray dataset measured in two different studies (S1 Text; there were 1,274 genes under F-test *p* < 0.01) [25,36].

### Clustering of Autism-Related Expression Changes

Genes sharing expression pattern difference in autism were identified using hierarchical clustering with (1-*r*), where *r* is the Pearson correlation coefficient, as the distance measure. The correlations were calculated based on expression profiles of control and autistic samples. We used the “complete” method of hierarchical clustering and cut the tree at a height from 2 to 0.3 (Fig 2A; S2 Fig). We discarded groups containing fewer than 40 genes. For each observed gene group under each height cutoff, the KEGG and GO functional enriched terms were calculated (S1 Text). The height cutoff = 1.4 yielded the highest enrichment of genes within clusters in functional GO terms and KEGG pathways (S2 Fig; S3 Data). To evaluate the robustness of autism-related expression patterns determined by the abovementioned procedure, we used the log2-transformation of RPKM values as gene expression level measurement or determined gene clusters based on all detected genes. These procedures resulted in 7 and 48 gene clusters, respectively, under the uniform tree height cutoff of 1.4 (S3 and S4 Figs). The clustering pattern enriched in synaptic genes, defined in our main analysis as cluster 2, remained robust using all described clustering procedures (S5 and S6 Figs).

### Identifying Genes with Species-Specific Expression

Genes with species-specific expression were identified as described in previous studies [36,37]. Briefly, the differential expression test, described in [67], was performed on each species pair twice, using either species as a reference. For each gene, if either of the two tests was significant at a defined cutoff, we considered this gene as differentially expressed between the two species. If a gene showed no significant expression differences between chimpanzees and macaques, but showed significant differences between humans and the other two primate species, this gene was assigned to the human-specific gene set. Chimpanzee-specific genes were defined using the analogous criteria. The FDR of the differential expression test was estimated as previously described [36]. Briefly, samples were divided into six age intervals, and the species’ identifiers were permuted within each group to preserve the age structure of the data. FDR was calculated by 1,000 random permutations of species identifiers.

The human-specific genes identified in this RNA-seq dataset overlapped significantly with the human-specific genes identified in the published microarray data (one-sided Fisher’s exact test, p < 0.0001) [36]. To identify human-specific expression changes supported by the current RNA-seq data as well as the published microarray data, we calculated the Pearson correlation between RNA-seq and microarray expression curves. The correlations were calculated based on expression levels of all three species interpolated at equal number of time points in each species, as previously described [36,37], to ensure equal power across species. Specifically, for each species, we interpolated expression values at 15 equally distributed points along the species’ age range shared between RNA-seq and microarray data at the (age)1/4 scale. We used cubic spline regression for interpolation, restricting the fit to three degrees of freedom. Human-specific genes identified in RNA-seq or microarray datasets with Pearson correlation coefficient between RNA-seq and microarray measurements greater than 0.5 were used as a consensus of human-specific expression gene set in the following analysis.

### Testing the Excess of Autism-Associated Genetic Mutations

To compare genes differentially expressed in autism with genes showing autism-associated genetic mutations, we used the following data: (1) 791 genes in SFARI AutDB database, which were extracted from studies on the molecular genetics and biology of ASD [38] (Updated in December, 2015; https://gene.sfari.org/autdb/Welcome.do; S7 Fig); (2) 375 scored genes with strong genetic evidence in the categories 1–4 in SFARI AutDB database (1 = high confidence, 2 = strong candidate, 3 = suggestive evidence, 4 = minimal evidence; Fig 2A); (3) 977 genes collected from the AutismKB database, which were restricted to genes exacted from the genome-wide association, linkage association, or low-scale association studies of autism spectrum disorder (ASD) [39] (http://autismkb.cbi.pku.edu.cn/; S7 Fig); (4) 1500 genes with de novo mutations associated with autism collected from four published whole-exome sequencing studies [8,10–12]. To test the excess of overlap between genes differentially expressed in autism with genes showing autism-associated genetic mutations, we calculated the expected-by-chance overlap by randomly sampling the same number of genes expressed in PFC as within the tested cluster, repeating the permutation 1,000 times.

To test the excess of autism-associated mutations in the potential regulators identified in cluster 2, we used the autism germline mutation index of genes calculated by whole-genome sequencing of monozygotic twins concordant for ASD and their parents from the study [50]. To calculate the significance of the excess of autism-related mutations in each gene, we compared the mutation index within the tested genes with the ones of genes randomly selected from the genome 1,000 times.

### TF Binding Site Enrichment Analysis

The identification of putative TF binding sites (TFBSs) was conducted as previously described [68]. Briefly, we identified the TFs showing enrichment of binding sites (TFBSs), in the regulatory region around the transcription start site of genes within one of the six clusters, and used hypergeometric test *p* < 0.05 as nominal cutoff. We used genes in the other five clusters as background. We further estimated the empirical significance of this result, and the randomly expected number of TFs showing the same level of TFBS enrichment, by repeating analysis on 1,000 random sets containing the same number of differentially expressed genes, sampled from all genes differentially expressed between autism patients and unaffected controls. The putative TFBSs were predicted using the Match algorithm [46] by scanning the TF consensus binding sequences from the TRANSFAC database in the promoter regions of each human Ensembl gene, defined as +/- 2 kb of the transcription start site (TSS). The predicted TFBSs were further restricted to those with ≥80% of nucleotides having defined phastCon score with the average ≥0.6 in the UCSC Genome Browser 17-way vertebrate Conserved Element table [69]. We thus defined 156 TFBSs and 192 corresponding TFs (TFBS-TF mapping was done based on the TRANSFAC binding site database). The correlated TFs and their potential targets were shown in S5 Data.

### TF-Target Correlation Analysis

We tested coexpression of TFs with enriched TFBS with their targets in the corresponding cluster as described elsewhere [36,37,68]. Briefly, based on each TF with enriched targets within a cluster, we calculated the Pearson’s correlation coefficients (PCCs) based on expression levels of TF and its targets within a cluster, and compared them with PCCs based on expression levels of TF and its targets in the other five clusters. We then compared the two PCC distributions using the Mann-Whitney U (MWU) test. The correlated TFs were defined as having BH corrected MWU test *p* < 0.05 for tests based on expression levels in autism and in control samples. To test whether positive correlations found between TFs and their predicted targets exceeded expectation, the expected-by-chance numbers of correlated TFs were calculated by randomly sampling the same number of autism differentially expressed genes within the tested cluster and repeating the enrichment test 1,000 times.

In total, there were four TFs, EGR1–4, showing significant binding site enrichment in cluster 2 genes, as well as significant expression profiles correlated with the expression of their predicted targets under *p* < 0.05 via BH corrected MWU test (Fig 4A). We have since tested the authenticity of the EGR target predictions using data of EGR1–2 ChIP-seq peaks from the ENCODE project [22] and two other studies [9,29]. We downloaded data from NCBI GEO, where the ChIP-seq experiments were done in ECC-1, GM12878, H1-hESC, HCT-116, K562, MCF-7, MAC, and LoVo cell lines (S6 Table). Data position coordinates of ChIP-seq peaks were converted to hg19 genome builds using UCSC LiftOver when necessary. The annotatePeak function in the R package “ChIPseeker” was used to link peaks with the nearest gene and genic region. For each ChIP-seq experiment, genes with peaks located within the promoter regions (+/- 2 kb of the TSSs) were considered as potential targets of the TF. Fisher’s exact test followed by BH correction for multiple testing was used to test the overlap of EGR targets predicted by searching TFBS sequence matrices from the TRANSFAC database in the promoter regions using Match algorithm and the targets predicted by ChIP-Seq experiments (S14 Fig).

## Supporting Information

S1 Data Gene expression levels (RPKM values) for all expressed genes. Genes are shown in rows. The sample group information (h: controls; a: autism cases; c: chimpanzees, m: macaques) and age in days are shown in columns.
(TXT)

S2 Data Dimensions 1 and 2 from the multidimensional scaling (MDS) of all expressed genes in autism cases and unaffected controls. (XLSX)

S3 Data Genes differently expressed between autism cases and unaffected controls. The cluster information, gene ensemble ID, and gene HGNC ID are shown in the first to third columns, respectively.
(TXT)

S4 Data Observed and expected gene numbers in Figs 2D, 3C and 4A. The expected numbers were estimated by 1,000 permutations of cluster labels.
(XLSX)

S5 Data The correlated TFs and their potential targets. The cluster information, correlated TFs, and their potential targets are shown in the first to third columns, respectively.
(TXT)

S1 Fig Correlation of expression differences between autism and control groups measured using RNA-seq and microarrays. The *x*-axis shows the mean expression difference between autism and control cases in the RNA-seq dataset, and the *y*-axis, the microarray dataset. The expression difference was calculated based on eight pairs of age-matched autism and control samples measured in both datasets (S1 Table). Each point represents expression difference for one gene; colors represent different gene sets (orange: genes with significant expression change in autism identified by reanalyzing the published microarray data; purple: genes with significant expression change in autism identified using the RNA-seq data; red: genes with significant expression change in autism identified in both datasets; blue: all other genes detected in both datasets). The inset numbers show the value of Pearson correlation coefficients for different gene sets.
(PDF)

S2 Fig The number and functional enrichment of clusters defined at different height cutoffs of the hierarchical clustering tree. (A) Hierarchical clustering of 1,775 genes differently expressed between autism cases and controls (the same as on Fig 2A). (B) Number of gene clusters obtained by cutting the hierarchical clustering tree at different heights. Clusters containing fewer than 40 genes were not counted. (C) The total number of GO functional terms and KEGG pathways significantly overrepresented among genes in each of the clusters identified using given tree cutting height cutoff. (D) Enrichment rank of GO functional terms and KEGG pathways determined for each height cutoff. The ranks are based on the total number of significantly enriched GO functional terms and KEGG pathways and were calculated using the rank function in R.
(PDF)

S3 Fig Gene clusters determined using log2-transformed RPKM values. The panels show expression patterns of the seven gene clusters of autism-related genes identified using log2-transformed RPKM values and uniform tree cutting cutoff at 1.4. The *x*-axis shows the age information on the (age)1/4 scale, the *y*-axis shows the expression levels standardized to mean = 0 and standard deviation = 1 before plotting. The points represent mean expression levels in each individual (red: controls; black: autism cases); the lines show cubic spline curves fitted to the individual data; the shaded areas show the standard deviation of the spline curves within a cluster. The cluster number and the number of genes within the cluster are shown on top of the panels.
(PDF)

S4 Fig Comparison of clusters based on the original and log2-transformed values. The cells show overlap between genes in six clusters used in the main analysis (*x*-axis) and genes in seven clusters obtained using log2-transformed RPKM values and uniform tree cutting cutoff at 1.4 (*y*-axis). Each cell shows the number and percentage of overlapping genes; the *p*-value indicating significance of the overlap calculated using Fisher's exact test followed by Benjamini-Hochberg (BH) correction for multiple testing.
(PDF)

S5 Fig Comparison of clusters based on 1,775 genes differentially expressed in autism (*x*-axis) and all 12,557 detected genes. The cells show overlap between genes in six clusters used in main analysis (*x*-axis) and genes in 48 clusters based on analysis of all 12,557 detected genes using RPKM values and uniform tree cutting cutoff at 1.4 (*y*-axis). Each cell shows the number and percentage of overlapping genes; the *p*-value indicating significance of the overlap calculated using Fisher’s exact test followed by Benjamini-Hochberg (BH) correction for multiple testing. The 48 clusters shown on the *y*-axis were sorted based on their BH-corrected *p*-values from the Fisher's exact test.
(PDF)

S6 Fig Enriched GO functional terms of cluster 4 (Call_4), overlapping with cluster 2 of the main analysis. GO functional terms significantly enriched in cluster 4 (Call_4) genes defined based on clustering of all genes detected as expressed in autism and control samples. The enriched functional terms (*y*-axis) were sorted based on the hypergeometric test *p*-values corrected for multiple testing using Benjamini-Hochberg (BH) correction. The *x*-axis shows–log10-transformated adjusted *p*-values of the enrichment test.
(PDF)

S7 Fig Profiles of expression changes in autism drawn for different levels of the disease phenotype. The symbols represent individual expression level measurements (red circles: controls; black stars: autism cases with high ADI-R scores; black triangles: autism cases with moderate ADI-R scores; black crosses: autism cases with low ADI-R scores). The lines represent cubic spline curves fitted to individual’s data (red: fitted to controls; gray: fitted to all autism cases; black: fitted to autism cases with moderate ADI-R scores). The *x*-axis shows age information and the *y*-axis shows the expression levels. Expression levels of all genes were standardized to mean = 0 and standard deviation = 1 before plotting. The titles on top of each panel show cluster information and the number of genes in each cluster.
(PDF)

S8 Fig Overlap between genes from six clusters representing major patterns of autism-related expression changes and genes associated with autism. The genes associated with autism were collected from: (A) the SFARI AutDB database, (B) the AutismKB database, (C) four published whole-exome sequencing studies. In each panel, the red bars show the actual numbers of overlapping genes, and the streaked bars show the mean number of overlapping genes expected by chance, estimated by 1,000 permutations of cluster labels. The symbols above the bars show the significance of the overlap, based on 1,000 permutations of cluster labels (***: p < 0.001; *: p < 0.05).
(PDF)

S9 Fig Gene length distribution in six major clusters of expression changes in autism. (A) The distribution for all genes in each cluster. (B) The distribution for genes sampled based on cluster 2 gene length distribution.
(PDF)

S10 Fig Overlap between genes associated with autism based on genetic studies and genes in six major clusters of expression changes in autism corrected for length difference as shown in S9 Fig. The genes associated with autism were collected from: (A) the SFARI AutDB database, (B) the SFARI scored genes, (C) the AutismKB database, (D) four published whole-exome sequencing studies. In each panel, the red bars show the actual numbers of overlapping genes, and the streaked bars show the mean number of overlapping genes expected by chance, estimated by 1,000 permutations of cluster labels. The symbols above the bars show the significance of the overlap, based on 1,000 permutations of cluster labels (***: *p* < 0.001; **: *p* < 0.01; *: *p* < 0.05).
(PDF)

S11 Fig Overlap of autism-related clusters (*x*-axis) and cell type markers collected from Zeisel et al, 2015 (*y*-axis). Each cell shows the number and percentage of overlapping genes; the *p*-value indicating significance of the overlap calculated using Fisher’s exact test followed by BH correction for multiple testing. The *y*-axis marker colors represent cell types (purple: neuron; orange: glia; blue: others).
(PDF)

S12 Fig Overlap between genes highly expressed in neurons and the six major clusters of expression changes in autism. The *y*-axis shows relative numbers of overlapping genes calculated as the log2 ratio between the observed gene number and the number expected by chance, calculated by 1,000 permutations of cluster labels. The error bars show the standard deviation of the fold-change estimates. The symbols above the bars show the significance of the overlap based on the 1,000 permutations (***: *p* < 0.001; **: *p* < 0.01; o: *p* < 0.1). Genes in cluster 5 are enriched in the marker genes of CCK-responsive (CCK+) neurons collected from Cahoy et al, 2008 (S3 Table).
(PDF)

S13 Fig Overlap of autism-related clusters (*x*-axis) and marker genes located in subcellular structures of neurons (*y*-axis). Marker genes located in presynapse, postsynapse, synaptic vesicle, axon, and dendrite collected based on GO cellular component annotation. Marker genes located in remaining neuron locations (category “Neuron” in the *y*-axis) were identified by excluding synapse, axon, and dendrite-related genes from neuron-related genes classified according to GO cellular component annotation. Each cell shows the number and percentage of overlapping genes; the *p*-value indicating significance of the overlap calculated using Fisher’s exact test followed by BH correction for multiple testing.
(PDF)

S14 Fig Overlap between six clusters used in the original analysis (*x*-axis) and macaque cortical layer marker genes for layers 2–5 (*y*-axis) collected from Bernard et al, 2012. Each cell shows the number and percentage of overlapping genes; the *p*-value indicating significance of the overlap calculated using Fisher's exact test followed by BH correction for multiple testing.
(PDF)

S15 Fig Correlation of age-related gene expression change between RNA-seq and microarray datasets. Shown are the distributions of Pearson correlation coefficients calculated based on expression levels at 15 points interpolated from the cubic spline curves fitted to individual microarray or RNA-seq expression measurements of each species (red: humans, blue: chimpanzees, green: macaque monkeys) or based on the expression levels interpolated using data from all three species (gray line). The distributions are based on 1,428 genes with human-specific developmental expression profiles in the PFC identified using microarray or RNA-seq data.
(PDF)

S16 Fig Expression profiles of pre- and postsynaptic marker genes, SYP and DLG4, in primate PFC. The boxes show median expression and the inter-quartile expression variation of SYP and DLG4 measured using RNA-seq. Colors represent different species (red: humans, blue: chimpanzees, green: macaque monkeys). To reduce inter-individual variation, individuals with similar ages were combined in seven age groups shown by dashed gray lines with age range indicated below (m: months, y: years).
(PDF)

S17 Fig Expression profiles of synapse-related genes measured by RNA-seq (left panel) or microarrays (right panel). The *x*-axis shows the age information on the (age)1/4 scale, the *y*-axis shows the expression levels standardized to mean = 0 and standard deviation = 1 before plotting. The points represent mean expression levels in each individual (red: controls; black: autism cases; blue: chimpanzees, green: macaques), the lines show cubic spline curves fitted to the individual data, and the error bars show standard deviation of the spline curves.
(PDF)

S18 Fig Distribution of Pearson correlation coefficients between gene expression and H3K4me3 modification differences in autism and control individuals. The expression difference or modification difference was calculated based on 8 pairs of age-matched autism and control samples in both datasets (S1 Table), with age ranged from 2 to 60 years old. The red lines represent the correlation coefficient distribution of genes in each cluster. The gray lines represent background distributions from other expressed genes. The cluster number and the significance of the positive correlation excess based on one-sided Wilcoxon test are shown on top of the cluster panels (***: *p* < 0.001; **: *p* < 0.01).
(PDF)

S19 Fig Comparison of computationally predicted and ChIP-seq-based EGR1/2 target genes. Cells show overlap of EGR1/2 target genes predicted using the TRANSFAC-based Match algorithm (“predict”) and identified using ChIP-seq data (S6 Table). Each cell shows the number and percentage of overlapping genes; the p-value indicating significance of the overlap calculated using Fisher's exact test followed by BH correction for multiple testing.
(PDF)

S20 Fig Distribution of mutations determined by the DNA sequence comparison between 10 monozygotic twins diagnosed with autism and their non-affected siblings. The red arrow indicates the mutation index in the four TF genes (EGR1-4) identified as potential regulators of the expression pattern detected in autism represented by the cluster 2. Note that EGR1 is enriched in mutations linked with autism (permutation test, *p* < 0.05 for EGR1).
(PDF)

S1 Table Sample information. (PDF)

S2 Table Summary of sequence reads in the RNA-seq batch. (PDF)

S3 Table Functional characteristics of genes in autism differentially expressed patterns. (PDF)

S4 Table Clinical information of autistic individuals used in this study. (PDF)

S5 Table Functional characteristics of overlapped genes between human-specific genes and autistic differentially expressed genes. (PDF)

S6 Table EGR target lists. (PDF)

S1 Text Supplementary methods. (PDF)

## Abbreviations

ADI-R: Autism Diagnostic Interview-Revised
ASD: autism spectrum disorder
EGR: early growth response
FDR: false discovery rate
GO: Gene Ontology
PFC: prefrontal cortex
TF: transcription factor
