# Supplementary figures and images for: Disruption of an Evolutionarily Novel Synaptic Expression Pattern in Autism
Source: PLoS Biol. 2016 Sep 29;14(9):e1002558. doi: 10.1371/journal.pbio.1002558 (PMC5042529; doi:10.1371/journal.pbio.1002558)

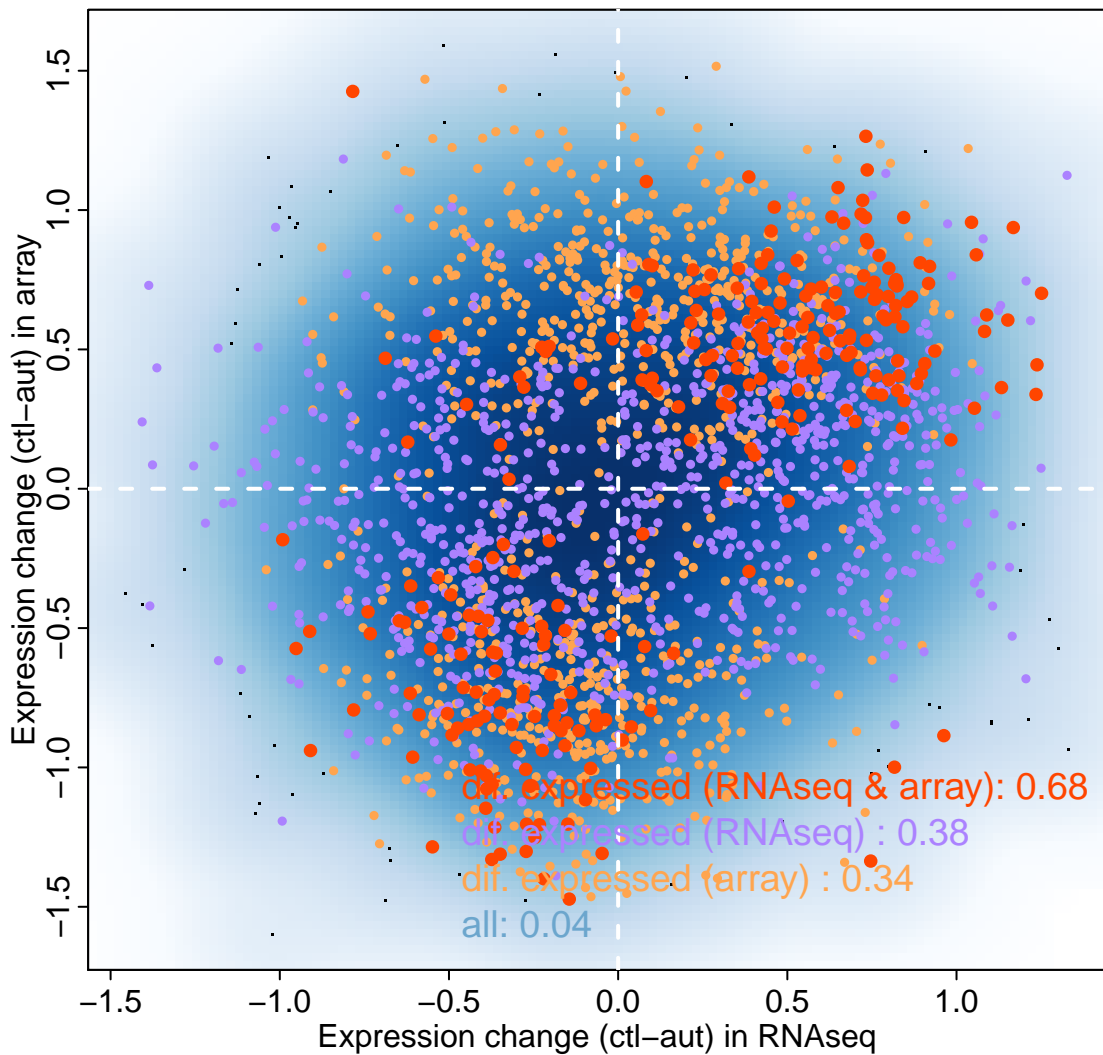

Supplement: S1 Fig — The x-axis shows the mean expression difference between autism and control cases in the RNA-seq dataset, and the y-axis, the microarray dataset. The expression difference was calculated based on eight pairs of age-matched autism and control samples measured in both datasets (S1 Table). Each point represents expression difference for one gene; colors represent different gene sets (orange: genes with significant expression change in autism identified by reanalyzing the published microarray data; purple: genes with significant expression change in autism identified using the RNA-seq data; red: genes with significant expression change in autism identified in both datasets; blue: all other genes detected in both datasets). The inset numbers show the value of Pearson correlation coefficients for different gene sets. (PDF) [file pbio.1002558.s006.pdf]

**A**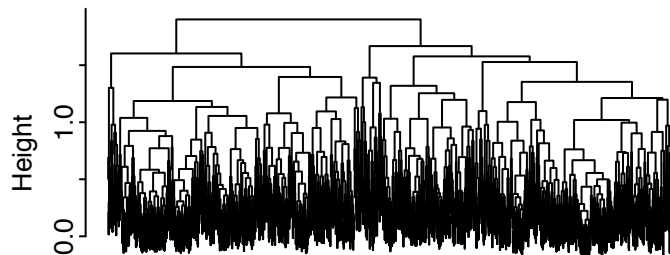**B**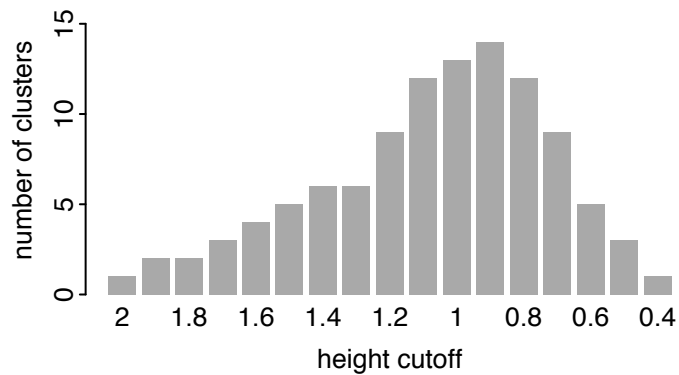**C**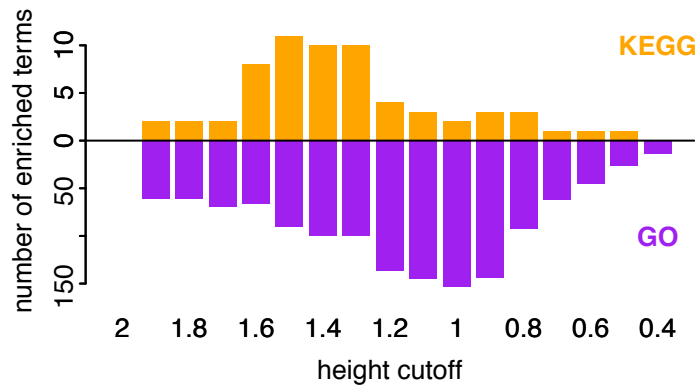**D**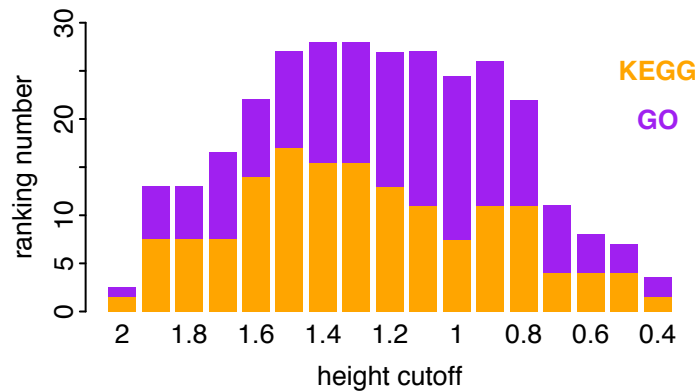

Supplement: S2 Fig — (A) Hierarchical clustering of 1,775 genes differently expressed between autism cases and controls (the same as on Fig 2A). (B) Number of gene clusters obtained by cutting the hierarchical clustering tree at different heights. Clusters containing fewer than 40 genes were not counted. (C) The total number of GO functional terms and KEGG pathways significantly overrepresented among genes in each of the clusters identified using given tree cutting height cutoff. (D) Enrichment rank of GO functional terms and KEGG pathways determined for each height cutoff. The ranks are based on the total number of significantly enriched GO functional terms and KEGG pathways and were calculated using the rank function in R. (PDF) [file pbio.1002558.s007.pdf]

**C\_log1:599**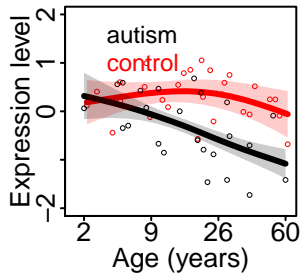**C\_log2:289**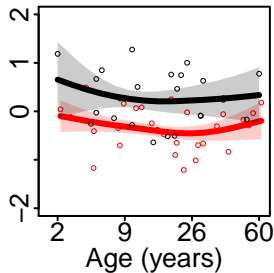**C\_log3:281**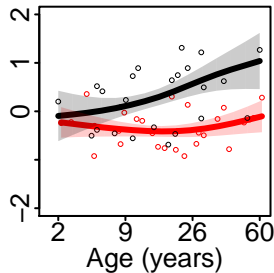**C\_log4:195**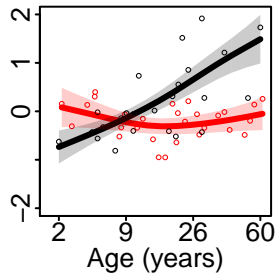**C\_log5:178**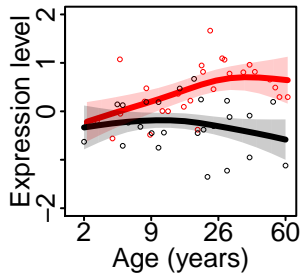**C\_log6:166**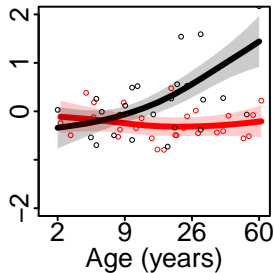**C\_log7:41**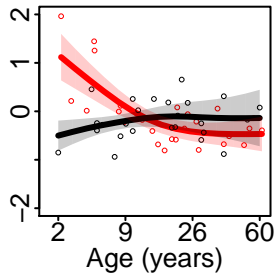

Supplement: S3 Fig — The panels show expression patterns of the seven gene clusters of autism-related genes identified using log2-transformed RPKM values and uniform tree cutting cutoff at 1.4. The x-axis shows the age information on the (age)1/4 scale, the y-axis shows the expression levels standardized to mean = 0 and standard deviation = 1 before plotting. The points represent mean expression levels in each individual (red: controls; black: autism cases); the lines show cubic spline curves fitted to the individual data; the shaded areas show the standard deviation of the spline curves within a cluster. The cluster number and the number of genes within the cluster are shown on top of the panels. (PDF) [file pbio.1002558.s008.pdf]

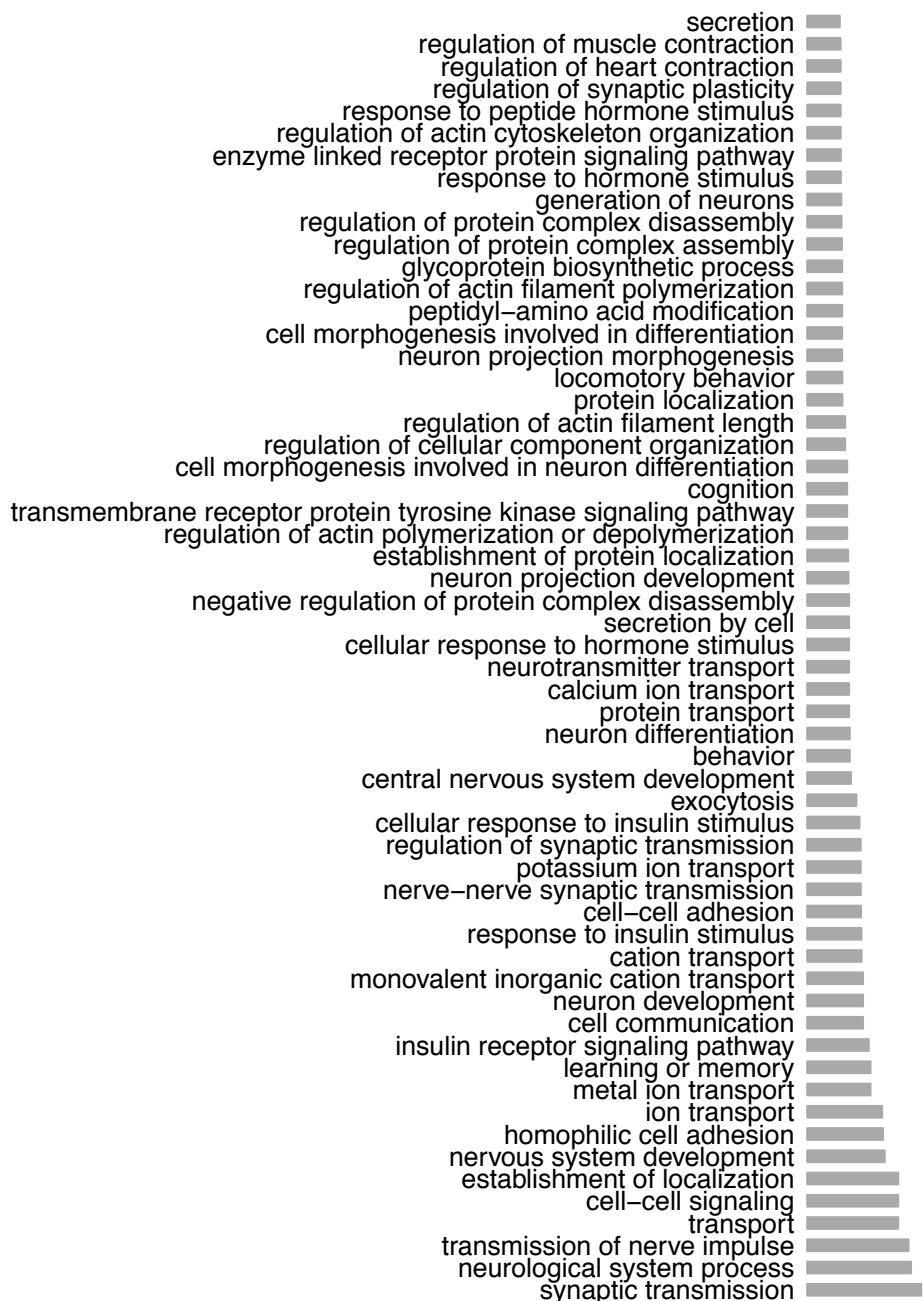

0 2 4

$-\log_{10}(\text{Adjusted P-values})$

Supplement: S6 Fig — GO functional terms significantly enriched in cluster 4 (Call_4) genes defined based on clustering of all genes detected as expressed in autism and control samples. The enriched functional terms (y-axis) were sorted based on the hypergeometric test p-values corrected for multiple testing using Benjamini-Hochberg (BH) correction. The x-axis shows–log10-transformated adjusted p-values of the enrichment test. (PDF) [file pbio.1002558.s011.pdf]

**C1:569**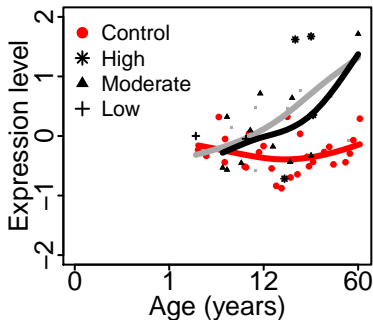**C2:454**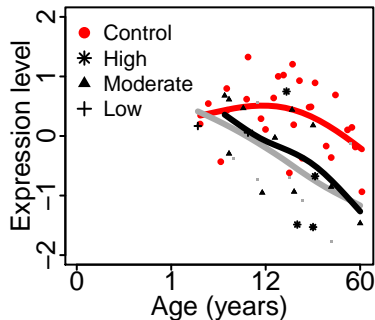**C3:308**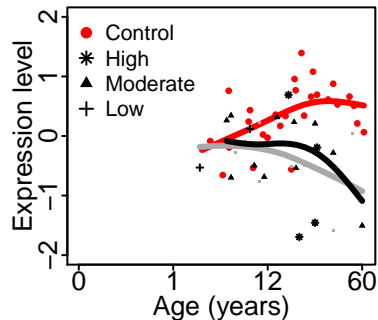**C4:293**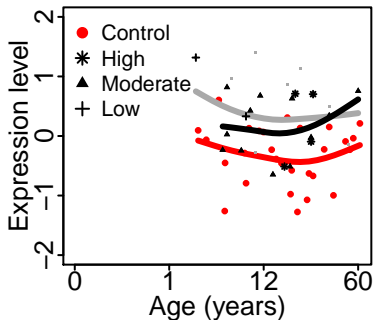**C5:66**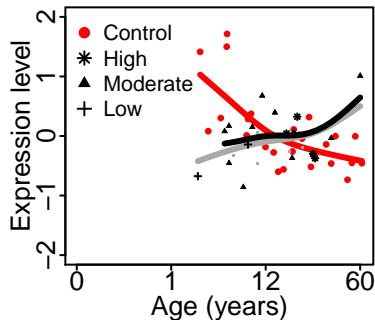**C6:50**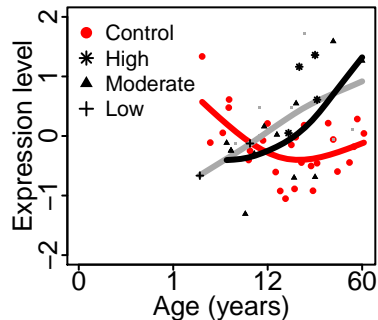

Supplement: S7 Fig — The symbols represent individual expression level measurements (red circles: controls; black stars: autism cases with high ADI-R scores; black triangles: autism cases with moderate ADI-R scores; black crosses: autism cases with low ADI-R scores). The lines represent cubic spline curves fitted to individual’s data (red: fitted to controls; gray: fitted to all autism cases; black: fitted to autism cases with moderate ADI-R scores). The x-axis shows age information and the y-axis shows the expression levels. Expression levels of all genes were standardized to mean = 0 and standard deviation = 1 before plotting. The titles on top of each panel show cluster information and the number of genes in each cluster. (PDF) [file pbio.1002558.s012.pdf]

**A** SFARI genes

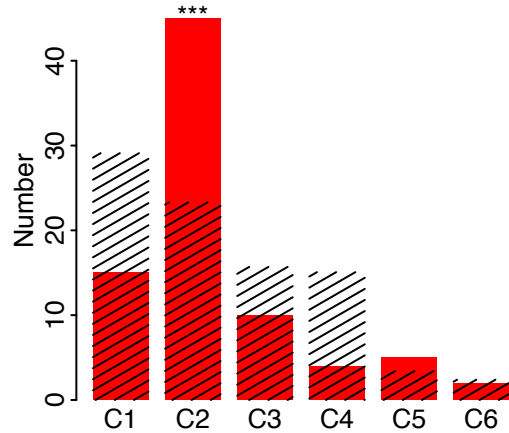

**B** AutismKB genes

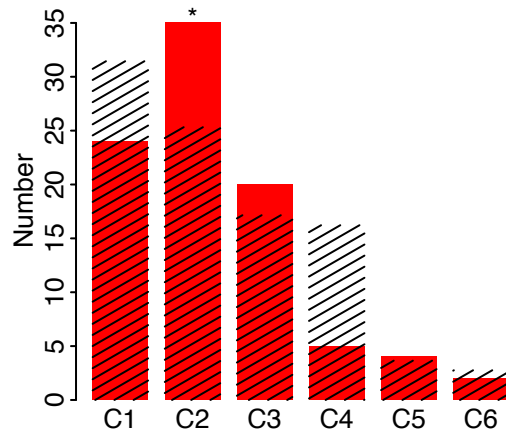

**C** Genes with *de novo* mutations in autism

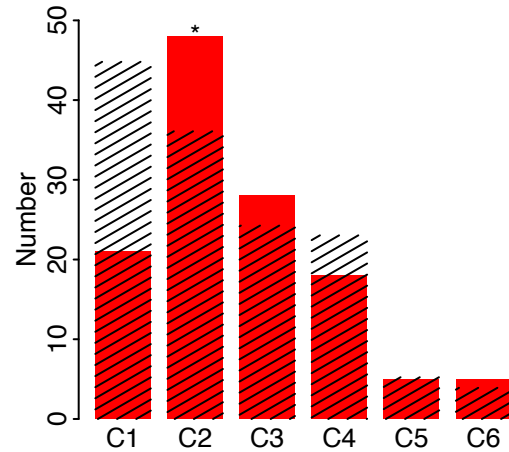

Supplement: S8 Fig — The genes associated with autism were collected from: (A) the SFARI AutDB database, (B) the AutismKB database, (C) four published whole-exome sequencing studies. In each panel, the red bars show the actual numbers of overlapping genes, and the streaked bars show the mean number of overlapping genes expected by chance, estimated by 1,000 permutations of cluster labels. The symbols above the bars show the significance of the overlap, based on 1,000 permutations of cluster labels (***: p < 0.001; *: p < 0.05). (PDF) [file pbio.1002558.s013.pdf]

**A****All**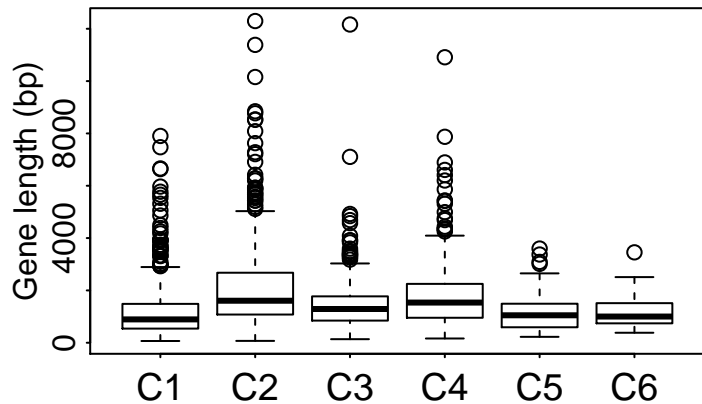**B****Subsamples**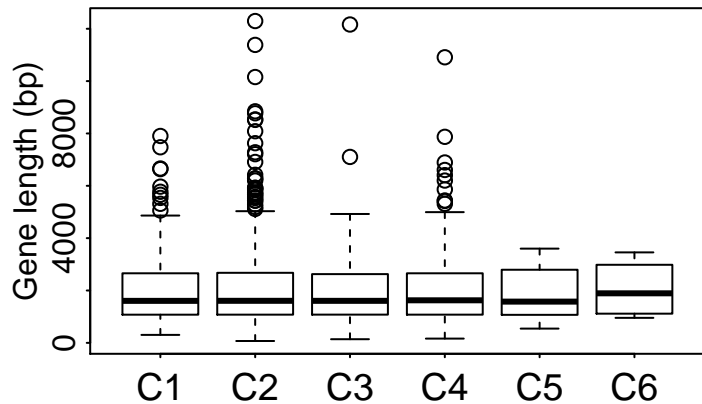

Supplement: S9 Fig — (A) The distribution for all genes in each cluster. (B) The distribution for genes sampled based on cluster 2 gene length distribution. (PDF) [file pbio.1002558.s014.pdf]

**A****SFARI genes**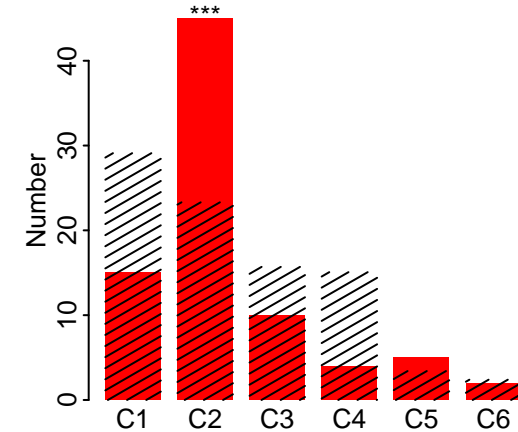**B****SFARI scored genes**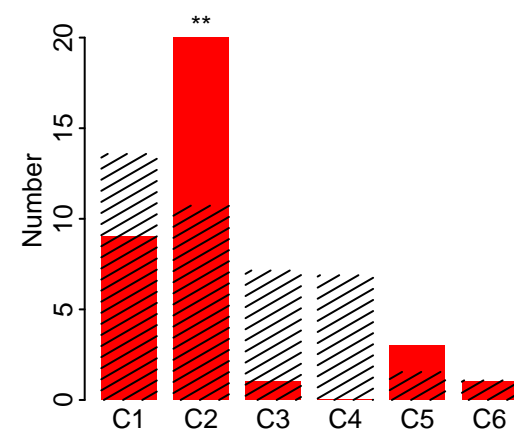**C****AutismKB genes**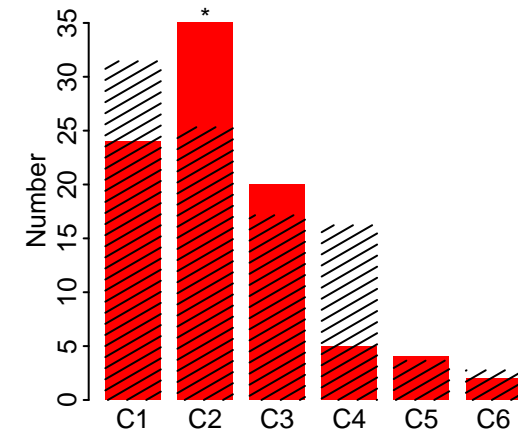**D****Genes with de novo mutations in autism**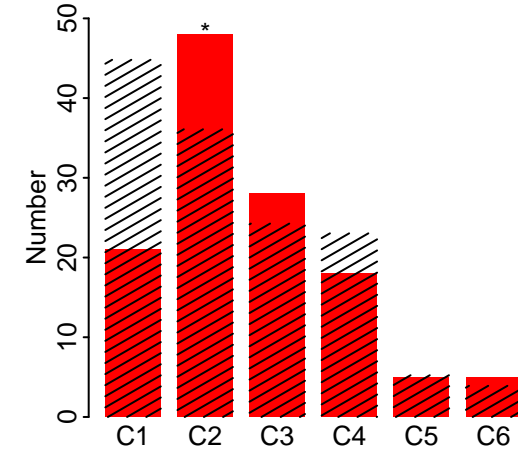

Supplement: S10 Fig — The genes associated with autism were collected from: (A) the SFARI AutDB database, (B) the SFARI scored genes, (C) the AutismKB database, (D) four published whole-exome sequencing studies. In each panel, the red bars show the actual numbers of overlapping genes, and the streaked bars show the mean number of overlapping genes expected by chance, estimated by 1,000 permutations of cluster labels. The symbols above the bars show the significance of the overlap, based on 1,000 permutations of cluster labels (***: p < 0.001; **: p < 0.01; *: p < 0.05). (PDF) [file pbio.1002558.s015.pdf]

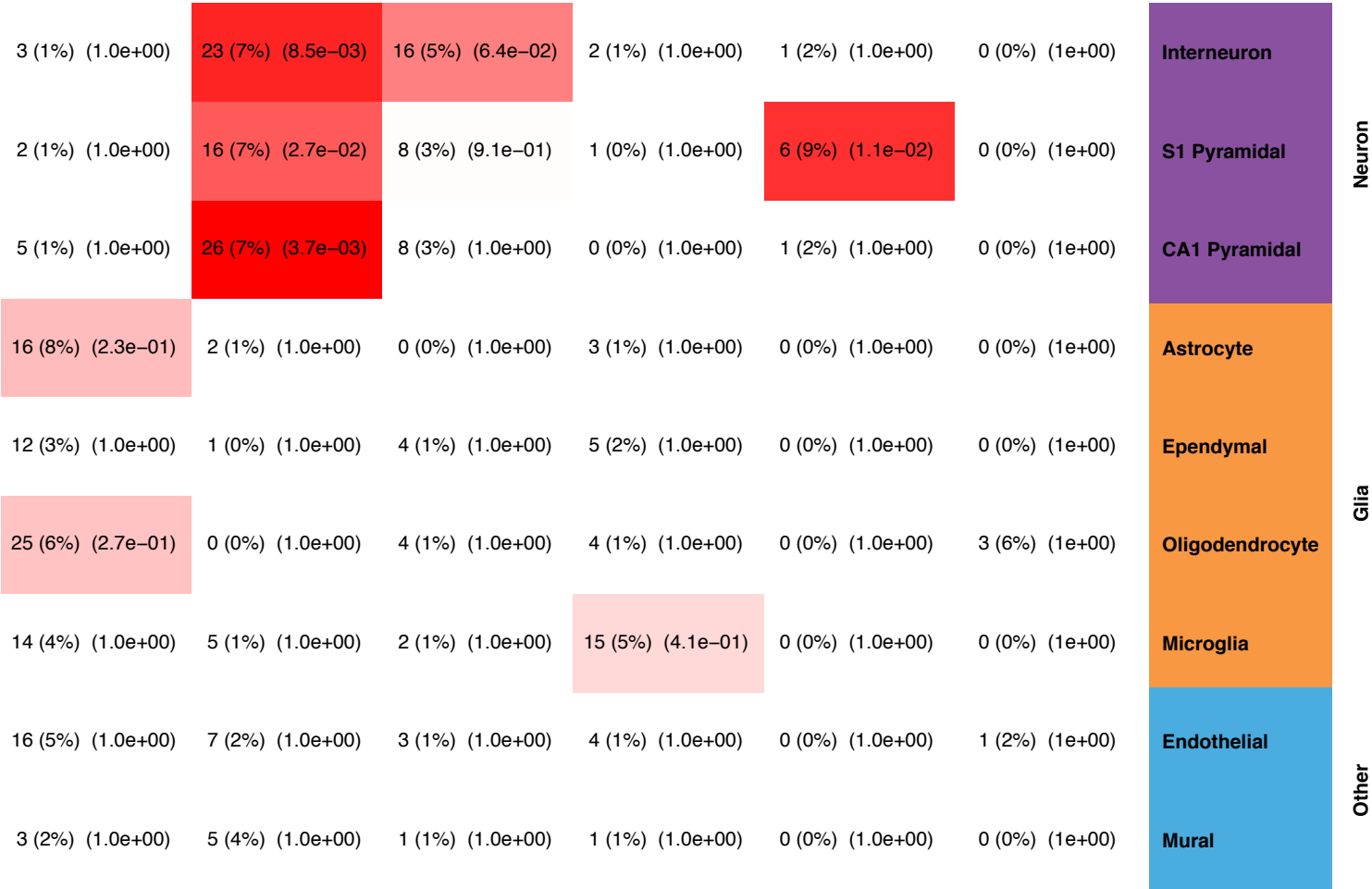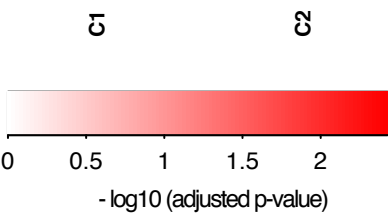

Supplement: S11 Fig — Each cell shows the number and percentage of overlapping genes; the p-value indicating significance of the overlap calculated using Fisher’s exact test followed by BH correction for multiple testing. The y-axis marker colors represent cell types (purple: neuron; orange: glia; blue: others). (PDF) [file pbio.1002558.s016.pdf]

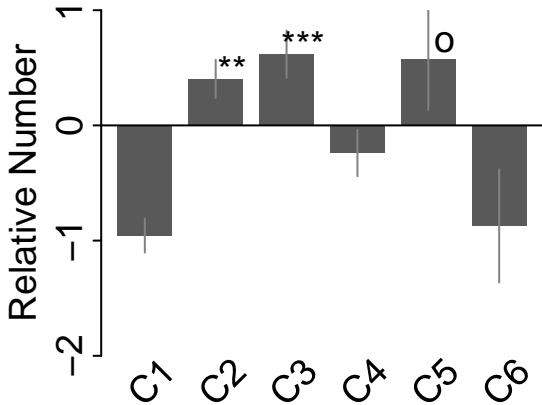

Supplement: S12 Fig — The y-axis shows relative numbers of overlapping genes calculated as the log2 ratio between the observed gene number and the number expected by chance, calculated by 1,000 permutations of cluster labels. The error bars show the standard deviation of the fold-change estimates. The symbols above the bars show the significance of the overlap based on the 1,000 permutations (***: p < 0.001; **: p < 0.01; o: p < 0.1). Genes in cluster 5 are enriched in the marker genes of CCK-responsive (CCK+) neurons collected from Cahoy et al, 2008 (S3 Table). (PDF) [file pbio.1002558.s017.pdf]

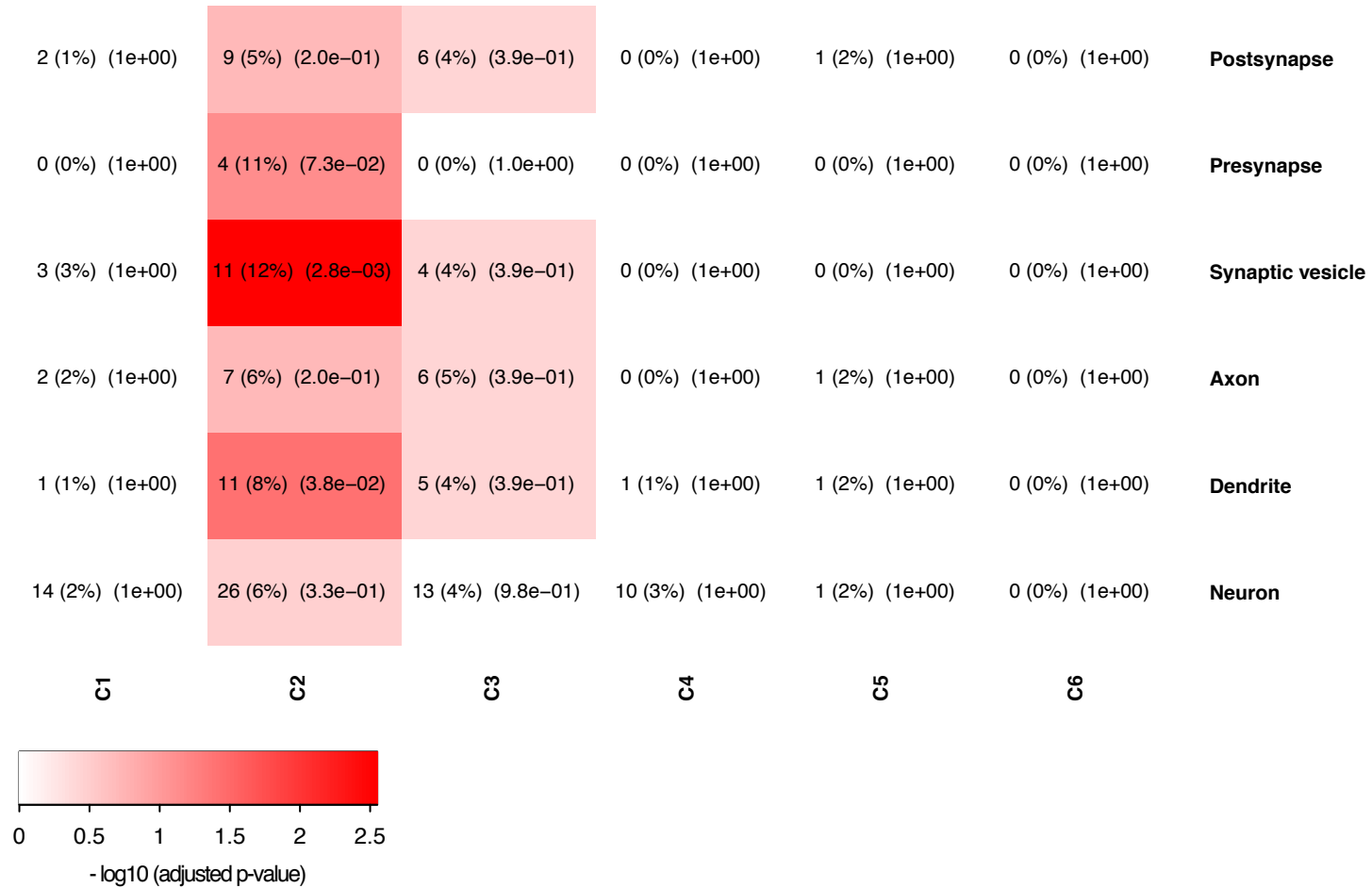

Supplement: S13 Fig — Marker genes located in presynapse, postsynapse, synaptic vesicle, axon, and dendrite collected based on GO cellular component annotation. Marker genes located in remaining neuron locations (category “Neuron” in the y-axis) were identified by excluding synapse, axon, and dendrite-related genes from neuron-related genes classified according to GO cellular component annotation. Each cell shows the number and percentage of overlapping genes; the p-value indicating significance of the overlap calculated using Fisher’s exact test followed by BH correction for multiple testing. (PDF) [file pbio.1002558.s018.pdf]

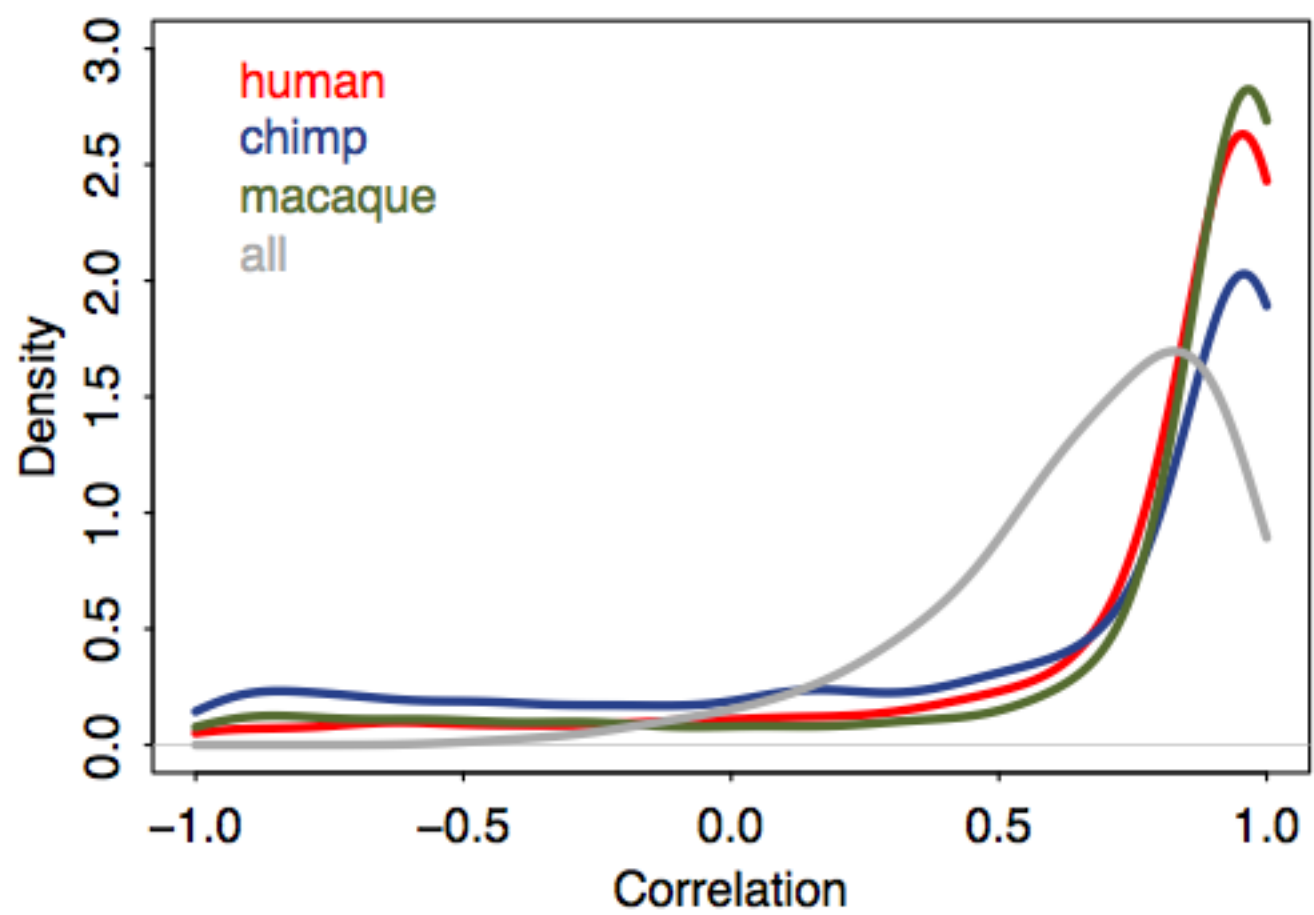

Supplement: S15 Fig — Shown are the distributions of Pearson correlation coefficients calculated based on expression levels at 15 points interpolated from the cubic spline curves fitted to individual microarray or RNA-seq expression measurements of each species (red: humans, blue: chimpanzees, green: macaque monkeys) or based on the expression levels interpolated using data from all three species (gray line). The distributions are based on 1,428 genes with human-specific developmental expression profiles in the PFC identified using microarray or RNA-seq data. (PDF) [file pbio.1002558.s020.pdf]

## SYP

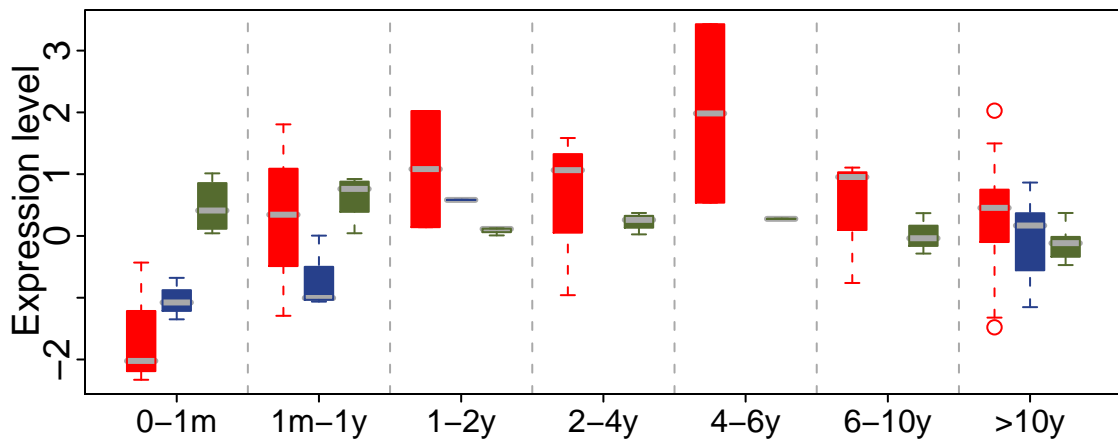

## DLG4

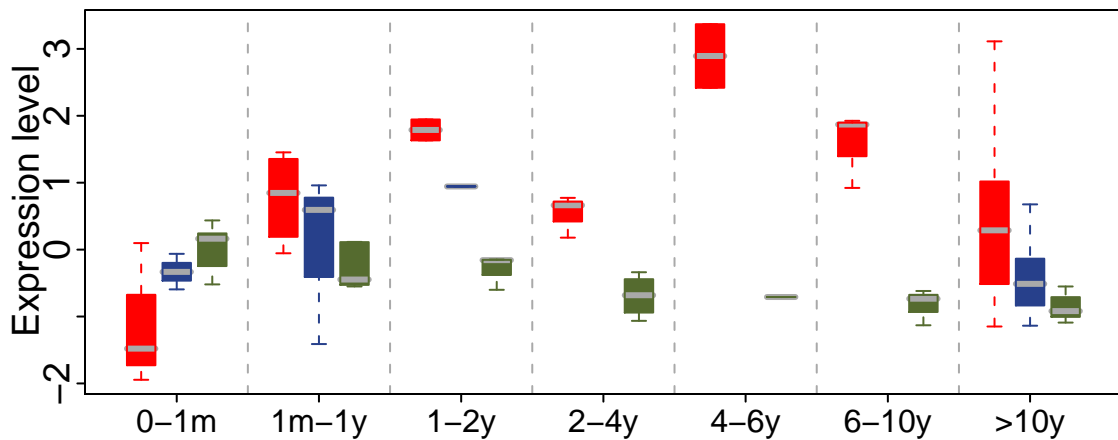

Supplement: S16 Fig — The boxes show median expression and the inter-quartile expression variation of SYP and DLG4 measured using RNA-seq. Colors represent different species (red: humans, blue: chimpanzees, green: macaque monkeys). To reduce inter-individual variation, individuals with similar ages were combined in seven age groups shown by dashed gray lines with age range indicated below (m: months, y: years). (PDF) [file pbio.1002558.s021.pdf]

**RNAseq (synapse-related genes)**

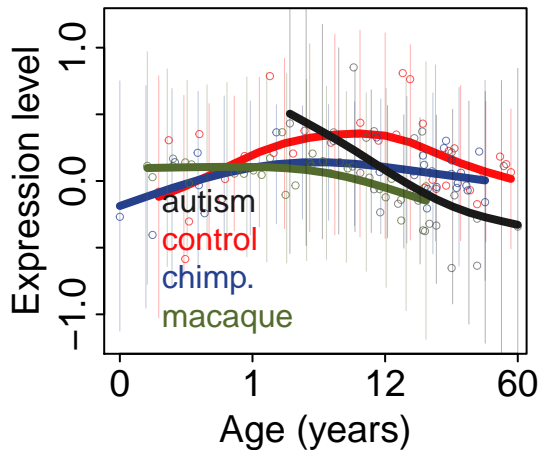

**Array (synapse-related genes)**

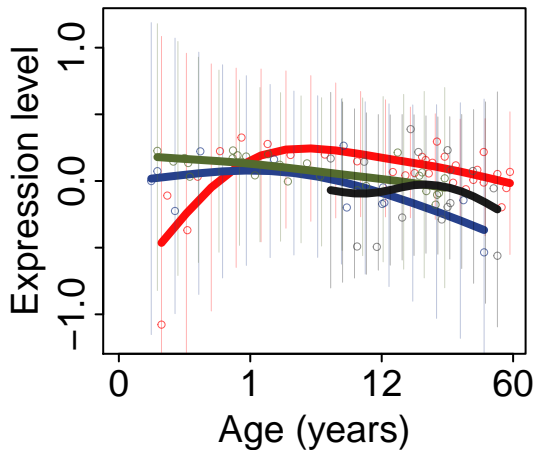

Supplement: S17 Fig — The x-axis shows the age information on the (age)1/4 scale, the y-axis shows the expression levels standardized to mean = 0 and standard deviation = 1 before plotting. The points represent mean expression levels in each individual (red: controls; black: autism cases; blue: chimpanzees, green: macaques), the lines show cubic spline curves fitted to the individual data, and the error bars show standard deviation of the spline curves. (PDF) [file pbio.1002558.s022.pdf]

**C1**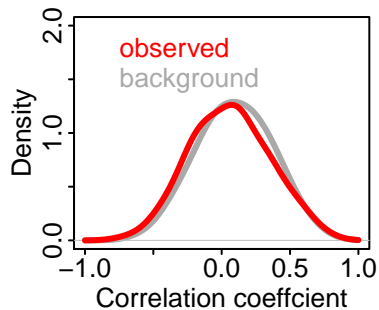**C2**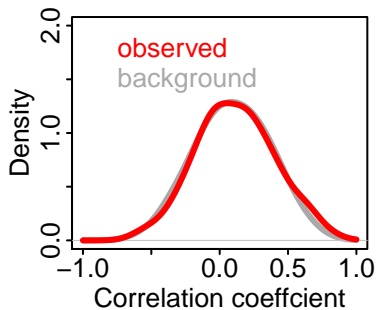**C3\*\*\***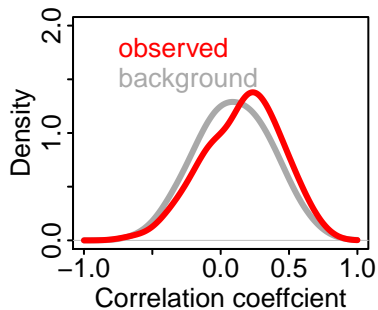**C4**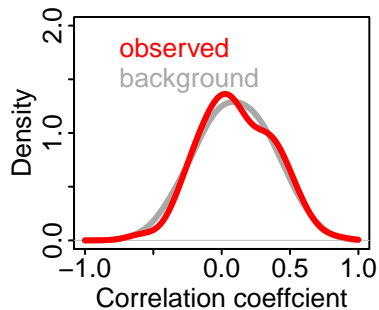**C5\*\***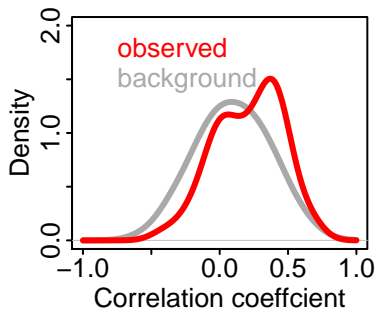**C6**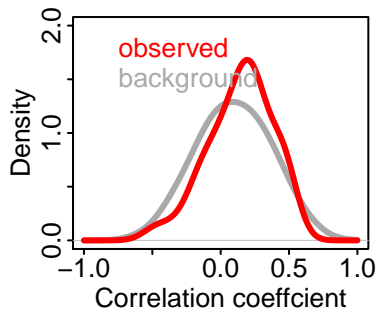

Supplement: S18 Fig — The expression difference or modification difference was calculated based on 8 pairs of age-matched autism and control samples in both datasets (S1 Table), with age ranged from 2 to 60 years old. The red lines represent the correlation coefficient distribution of genes in each cluster. The gray lines represent background distributions from other expressed genes. The cluster number and the significance of the positive correlation excess based on one-sided Wilcoxon test are shown on top of the cluster panels (***: p < 0.001; **: p < 0.01). (PDF) [file pbio.1002558.s023.pdf]

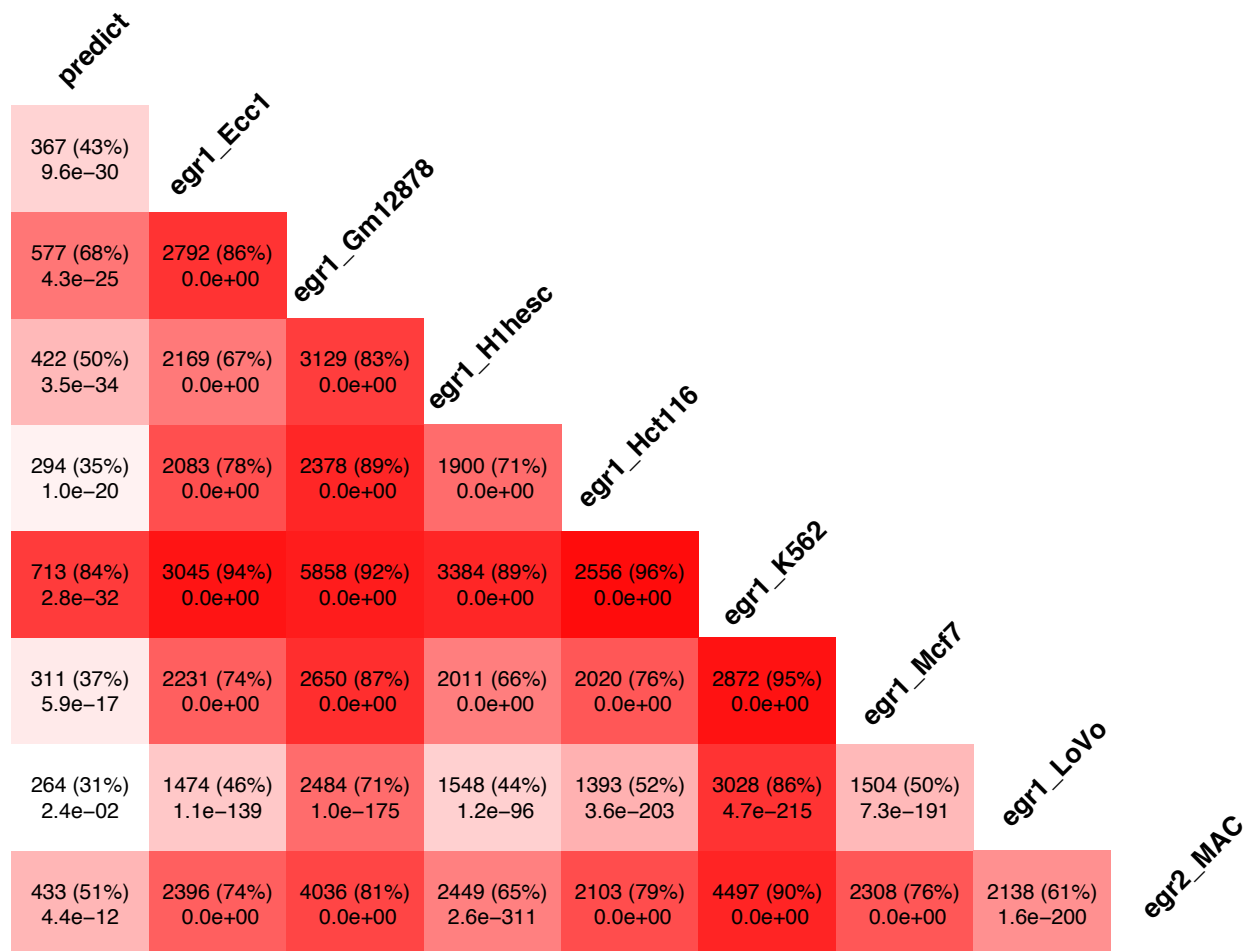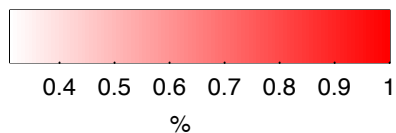

Supplement: S19 Fig — Cells show overlap of EGR1/2 target genes predicted using the TRANSFAC-based Match algorithm (“predict”) and identified using ChIP-seq data (S6 Table). Each cell shows the number and percentage of overlapping genes; the p-value indicating significance of the overlap calculated using Fisher's exact test followed by BH correction for multiple testing. (PDF) [file pbio.1002558.s024.pdf]

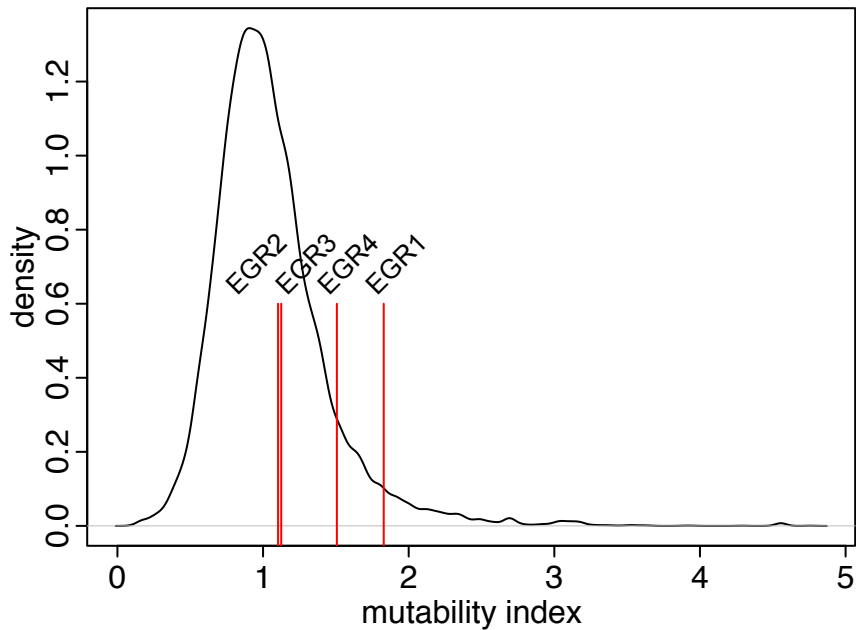

Supplement: S20 Fig — The red arrow indicates the mutation index in the four TF genes (EGR1-4) identified as potential regulators of the expression pattern detected in autism represented by the cluster 2. Note that EGR1 is enriched in mutations linked with autism (permutation test, p < 0.05 for EGR1). (PDF) [file pbio.1002558.s025.pdf]
