# Supplementary material for: Disruption of an Evolutionarily Novel Synaptic Expression Pattern in Autism
Source: PLoS Biol. 2016 Sep 29;14(9):e1002558. doi: 10.1371/journal.pbio.1002558 (PMC5042529; doi:10.1371/journal.pbio.1002558)
Supplement: S4 Fig — The cells show overlap between genes in six clusters used in the main analysis (x-axis) and genes in seven clusters obtained using log2-transformed RPKM values and uniform tree cutting cutoff at 1.4 (y-axis). Each cell shows the number and percentage of overlapping genes; the p-value indicating significance of the overlap calculated using Fisher's exact test followed by Benjamini-Hochberg (BH) correction for multiple testing. (PDF) [file pbio.1002558.s009.pdf]

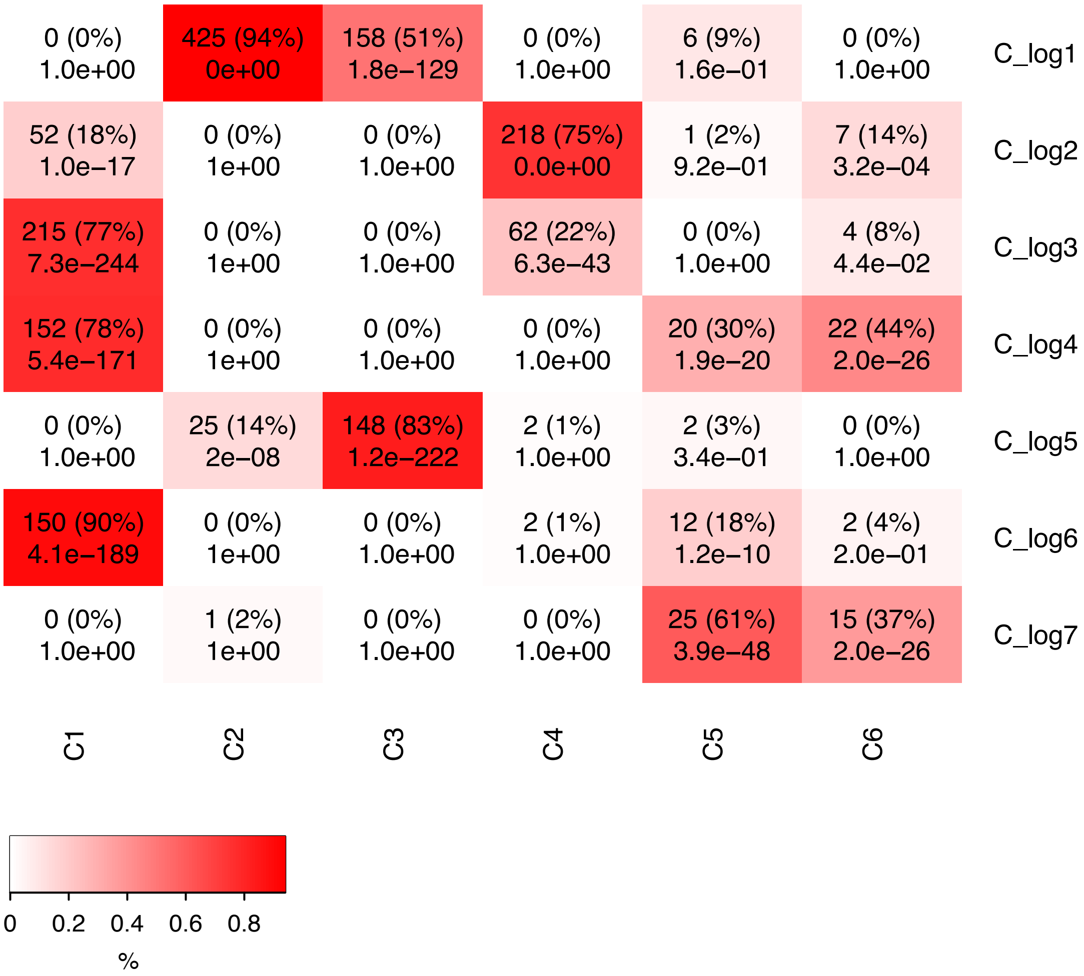
0 (0%) 1.0e+00
425 (94%) 0e+00
158 (51%) 1.8e−129
0 (0%) 1.0e+00
6 (9%) 1.6e−01
0 (0%) 1.0e+00
C_log1
52 (18%) 1.0e−17
0 (0%) 1e+00
0 (0%) 1.0e+00
218 (75%) 0.0e+00
1 (2%) 9.2e−01
7 (14%) 3.2e−04
C_log2
215 (77%) 7.3e−244
0 (0%) 1e+00
0 (0%) 1.0e+00
62 (22%) 6.3e−43
0 (0%) 1.0e+00
4 (8%) 4.4e−02
C_log3
152 (78%) 5.4e−171
0 (0%) 1e+00
0 (0%) 1.0e+00
0 (0%) 1.0e+00
20 (30%) 1.9e−20
22 (44%) 2.0e−26
C_log4
0 (0%) 1.0e+00
25 (14%) 2e−08
148 (83%) 1.2e−222
2 (1%) 1.0e+00
2 (3%) 3.4e−01
0 (0%) 1.0e+00
C_log5
150 (90%) 4.1e−189
0 (0%) 1e+00
0 (0%) 1.0e+00
2 (1%) 1.0e+00
12 (18%) 1.2e−10
2 (4%) 2.0e−01
C_log6
0 (0%) 1.0e+00
1 (2%) 1e+00
0 (0%) 1.0e+00
0 (0%) 1.0e+00
25 (61%) 3.9e−48
15 (37%) 2.0e−26
C_log7
C1
C2
C3
C4
C5
C6
0
0.2
0.4
0.6
0.8
%
