# Supplementary material for: Disruption of an Evolutionarily Novel Synaptic Expression Pattern in Autism
Source: PLoS Biol. 2016 Sep 29;14(9):e1002558. doi: 10.1371/journal.pbio.1002558 (PMC5042529; doi:10.1371/journal.pbio.1002558)
Supplement: S5 Fig — The cells show overlap between genes in six clusters used in main analysis (x-axis) and genes in 48 clusters based on analysis of all 12,557 detected genes using RPKM values and uniform tree cutting cutoff at 1.4 (y-axis). Each cell shows the number and percentage of overlapping genes; the p-value indicating significance of the overlap calculated using Fisher’s exact test followed by Benjamini-Hochberg (BH) correction for multiple testing. The 48 clusters shown on the y-axis were sorted based on their BH-corrected p-values from the Fisher's exact test. (PDF) [file pbio.1002558.s010.pdf]

	C1	C2	C3	C4	C5	C6
C_all4	0 (0%) (1.0e+00)	195 (43%) (1.3e−159)	17 (6%) (6.7e−01)	0 (0%) (1.0e+00)	1 (2%) (1.0e+00)	0 (0%) (1.0e+00)
C_all14	97 (31%) (2.4e−53)	0 (0%) (1.0e+00)	0 (0%) (1.0e+00)	0 (0%) (1.0e+00)	4 (6%) (4.5e−01)	0 (0%) (1.0e+00)
C_all11	100 (29%) (4.9e−53)	0 (0%) (1.0e+00)	0 (0%) (1.0e+00)	0 (0%) (1.0e+00)	1 (2%) (1.0e+00)	3 (6%) (9.4e−01)
C_all20	0 (0%) (1.0e+00)	53 (19%) (6.9e−23)	71 (26%) (1.4e−51)	0 (0%) (1.0e+00)	0 (0%) (1.0e+00)	0 (0%) (1.0e+00)
C_all17	0 (0%) (1.0e+00)	63 (21%) (3.8e−30)	70 (24%) (1.0e−48)	0 (0%) (1.0e+00)	2 (3%) (1.0e+00)	4 (8%) (2.3e−01)
C_all29	0 (0%) (1.0e+00)	6 (3%) (1.0e+00)	44 (24%) (2.5e−30)	0 (0%) (1.0e+00)	0 (0%) (1.0e+00)	0 (0%) (1.0e+00)
C_all10	71 (20%) (2.9e−26)	0 (0%) (1.0e+00)	0 (0%) (1.0e+00)	0 (0%) (1.0e+00)	1 (2%) (1.0e+00)	5 (10%) (1.2e−01)
C_all2	5 (1%) (1.0e+00)	0 (0%) (1.0e+00)	2 (1%) (1.0e+00)	80 (27%) (4.1e−26)	0 (0%) (1.0e+00)	2 (4%) (1.0e+00)
C_all26	55 (25%) (1.1e−24)	0 (0%) (1.0e+00)	0 (0%) (1.0e+00)	0 (0%) (1.0e+00)	2 (3%) (1.0e+00)	0 (0%) (1.0e+00)
C_all15	3 (1%) (1.0e+00)	0 (0%) (1.0e+00)	0 (0%) (1.0e+00)	39 (13%) (1.1e−16)	0 (0%) (1.0e+00)	0 (0%) (1.0e+00)
C_all23	35 (15%) (6.1e−09)	0 (0%) (1.0e+00)	0 (0%) (1.0e+00)	34 (14%) (3.1e−16)	0 (0%) (1.0e+00)	0 (0%) (1.0e+00)
C_all31	0 (0%) (1.0e+00)	9 (6%) (8.2e−01)	27 (18%) (6.2e−15)	0 (0%) (1.0e+00)	0 (0%) (1.0e+00)	0 (0%) (1.0e+00)
C_all44	0 (0%) (1.0e+00)	0 (0%) (1.0e+00)	0 (0%) (1.0e+00)	0 (0%) (1.0e+00)	13 (20%) (2.1e−14)	0 (0%) (1.0e+00)
C_all1	0 (0%) (1.0e+00)	91 (20%) (3.6e−13)	2 (1%) (1.0e+00)	0 (0%) (1.0e+00)	0 (0%) (1.0e+00)	0 (0%) (1.0e+00)
C_all8	2 (1%) (1.0e+00)	0 (0%) (1.0e+00)	0 (0%) (1.0e+00)	36 (12%) (2.9e−12)	0 (0%) (1.0e+00)	0 (0%) (1.0e+00)
C_all9	36 (10%) (3.0e−05)	1 (0%) (1.0e+00)	0 (0%) (1.0e+00)	34 (12%) (2.1e−11)	0 (0%) (1.0e+00)	0 (0%) (1.0e+00)
C_all27	0 (0%) (1.0e+00)	0 (0%) (1.0e+00)	26 (12%) (3.3e−10)	0 (0%) (1.0e+00)	1 (2%) (1.0e+00)	0 (0%) (1.0e+00)
C_all7	50 (12%) (3.3e−09)	0 (0%) (1.0e+00)	0 (0%) (1.0e+00)	34 (12%) (2.2e−09)	1 (2%) (1.0e+00)	11 (22%) (1.5e−05)
C_all6	51 (12%) (3.0e−09)	0 (0%) (1.0e+00)	0 (0%) (1.0e+00)	0 (0%) (1.0e+00)	0 (0%) (1.0e+00)	8 (16%) (4.5e−03)
C_all39	0 (0%) (1.0e+00)	0 (0%) (1.0e+00)	0 (0%) (1.0e+00)	0 (0%) (1.0e+00)	3 (5%) (1.1e−01)	7 (14%) (6.0e−06)
C_all47	0 (0%) (1.0e+00)	1 (2%) (1.0e+00)	0 (0%) (1.0e+00)	0 (0%) (1.0e+00)	6 (9%) (2.6e−05)	0 (0%) (1.0e+00)
C_all45	0 (0%) (1.0e+00)	0 (0%) (1.0e+00)	0 (0%) (1.0e+00)	0 (0%) (1.0e+00)	6 (9%) (8.5e−05)	0 (0%) (1.0e+00)
C_all46	1 (1%) (1.0e+00)	0 (0%) (1.0e+00)	0 (0%) (1.0e+00)	1 (1%) (1.0e+00)	5 (8%) (5.0e−04)	1 (2%) (1.0e+00)
C_all18	27 (9%) (1.6e−03)	0 (0%) (1.0e+00)	0 (0%) (1.0e+00)	6 (2%) (1.0e+00)	0 (0%) (1.0e+00)	0 (0%) (1.0e+00)
C_all33	16 (12%) (1.6e−03)	0 (0%) (1.0e+00)	0 (0%) (1.0e+00)	2 (2%) (1.0e+00)	0 (0%) (1.0e+00)	1 (2%) (1.0e+00)
C_all24	0 (0%) (1.0e+00)	0 (0%) (1.0e+00)	6 (3%) (1.0e+00)	0 (0%) (1.0e+00)	6 (9%) (1.4e−02)	3 (6%) (4.7e−01)
C_all12	0 (0%) (1.0e+00)	6 (2%) (1.0e+00)	2 (1%) (1.0e+00)	0 (0%) (1.0e+00)	7 (11%) (1.5e−02)	0 (0%) (1.0e+00)
C_all25	0 (0%) (1.0e+00)	0 (0%) (1.0e+00)	1 (0%) (1.0e+00)	0 (0%) (1.0e+00)	0 (0%) (1.0e+00)	5 (10%) (2.3e−02)
C_all38	0 (0%) (1.0e+00)	0 (0%) (1.0e+00)	8 (8%) (2.7e−02)	0 (0%) (1.0e+00)	0 (0%) (1.0e+00)	0 (0%) (1.0e+00)
C_all19	0 (0%) (1.0e+00)	18 (6%) (1.2e−01)	5 (2%) (1.0e+00)	0 (0%) (1.0e+00)	0 (0%) (1.0e+00)	0 (0%) (1.0e+00)
C_all41	1 (1%) (1.0e+00)	0 (0%) (1.0e+00)	6 (7%) (1.4e−01)	1 (1%) (1.0e+00)	0 (0%) (1.0e+00)	0 (0%) (1.0e+00)
C_all28	0 (0%) (1.0e+00)	1 (1%) (1.0e+00)	0 (0%) (1.0e+00)	0 (0%) (1.0e+00)	3 (5%) (4.5e−01)	0 (0%) (1.0e+00)
C_all34	6 (5%) (1.0e+00)	0 (0%) (1.0e+00)	0 (0%) (1.0e+00)	6 (5%) (4.7e−01)	0 (0%) (1.0e+00)	0 (0%) (1.0e+00)
C_all3	0 (0%) (1.0e+00)	1 (0%) (1.0e+00)	3 (1%) (1.0e+00)	6 (2%) (1.0e+00)	0 (0%) (1.0e+00)	0 (0%) (1.0e+00)
C_all5	1 (0%) (1.0e+00)	2 (0%) (1.0e+00)	1 (0%) (1.0e+00)	6 (2%) (1.0e+00)	0 (0%) (1.0e+00)	0 (0%) (1.0e+00)
C_all13	0 (0%) (1.0e+00)	0 (0%) (1.0e+00)	0 (0%) (1.0e+00)	2 (1%) (1.0e+00)	0 (0%) (1.0e+00)	0 (0%) (1.0e+00)
C_all16	4 (1%) (1.0e+00)	0 (0%) (1.0e+00)	6 (2%) (1.0e+00)	0 (0%) (1.0e+00)	3 (5%) (1.0e+00)	0 (0%) (1.0e+00)
C_all21	0 (0%) (1.0e+00)	0 (0%) (1.0e+00)	2 (1%) (1.0e+00)	2 (1%) (1.0e+00)	0 (0%) (1.0e+00)	0 (0%) (1.0e+00)
C_all22	1 (0%) (1.0e+00)	2 (1%) (1.0e+00)	0 (0%) (1.0e+00)	0 (0%) (1.0e+00)	0 (0%) (1.0e+00)	0 (0%) (1.0e+00)
C_all30	0 (0%) (1.0e+00)	0 (0%) (1.0e+00)	1 (1%) (1.0e+00)	1 (1%) (1.0e+00)	1 (2%) (1.0e+00)	0 (0%) (1.0e+00)
C_all32	0 (0%) (1.0e+00)	1 (1%) (1.0e+00)	1 (1%) (1.0e+00)	3 (2%) (1.0e+00)	0 (0%) (1.0e+00)	0 (0%) (1.0e+00)
C_all35	2 (2%) (1.0e+00)	0 (0%) (1.0e+00)	0 (0%) (1.0e+00)	0 (0%) (1.0e+00)	0 (0%) (1.0e+00)	0 (0%) (1.0e+00)
C_all36	3 (3%) (1.0e+00)	0 (0%) (1.0e+00)	2 (2%) (1.0e+00)	0 (0%) (1.0e+00)	0 (0%) (1.0e+00)	0 (0%) (1.0e+00)
C_all37	0 (0%) (1.0e+00)	0 (0%) (1.0e+00)	2 (2%) (1.0e+00)	0 (0%) (1.0e+00)	0 (0%) (1.0e+00)	0 (0%) (1.0e+00)
C_all40	0 (0%) (1.0e+00)	1 (1%) (1.0e+00)	3 (3%) (1.0e+00)	0 (0%) (1.0e+00)	0 (0%) (1.0e+00)	0 (0%) (1.0e+00)
C_all42	2 (2%) (1.0e+00)	0 (0%) (1.0e+00)	0 (0%) (1.0e+00)	0 (0%) (1.0e+00)	0 (0%) (1.0e+00)	0 (0%) (1.0e+00)
C_all43	0 (0%) (1.0e+00)	2 (2%) (1.0e+00)	0 (0%) (1.0e+00)	0 (0%) (1.0e+00)	0 (0%) (1.0e+00)	0 (0%) (1.0e+00)
C_all48	0 (0%) (1.0e+00)	1 (2%) (1.0e+00)	0 (0%) (1.0e+00)	0 (0%) (1.0e+00)	0 (0%) (1.0e+00)	0 (0%) (1.0e+00)

0 0.1 0.2 0.3 0.4

%
