# Supplementary material for: Disruption of an Evolutionarily Novel Synaptic Expression Pattern in Autism
Source: PLoS Biol. 2016 Sep 29;14(9):e1002558. doi: 10.1371/journal.pbio.1002558 (PMC5042529; doi:10.1371/journal.pbio.1002558)
Supplement: S14 Fig — Each cell shows the number and percentage of overlapping genes; the p-value indicating significance of the overlap calculated using Fisher's exact test followed by BH correction for multiple testing. (PDF) [file pbio.1002558.s019.pdf]

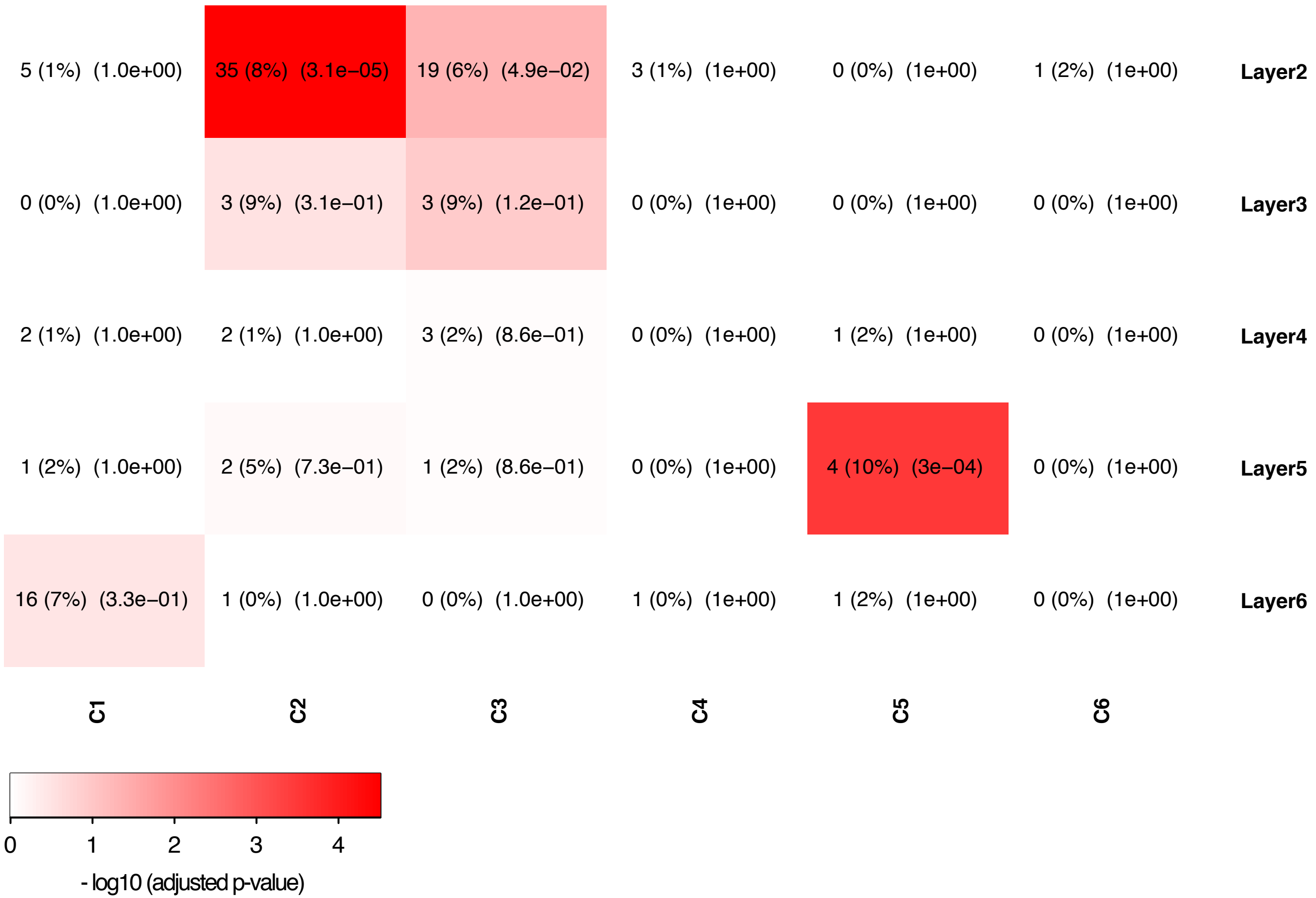

5 (1%) (1.0e+00)
35 (8%) (3.1e−05)
19 (6%) (4.9e−02)
3 (1%) (1e+00)
0 (0%) (1e+00)
1 (2%) (1e+00)
Layer2
0 (0%) (1.0e+00)
3 (9%) (3.1e−01)
3 (9%) (1.2e−01)
0 (0%) (1e+00)
0 (0%) (1e+00)
0 (0%) (1e+00)
Layer3
2 (1%) (1.0e+00)
2 (1%) (1.0e+00)
3 (2%) (8.6e−01)
0 (0%) (1e+00)
1 (2%) (1e+00)
0 (0%) (1e+00)
Layer4
1 (2%) (1.0e+00)
2 (5%) (7.3e−01)
1 (2%) (8.6e−01)
0 (0%) (1e+00)
4 (10%) (3e−04)
0 (0%) (1e+00)
Layer5
16 (7%) (3.3e−01)
1 (0%) (1.0e+00)
0 (0%) (1.0e+00)
1 (0%) (1e+00)
1 (2%) (1e+00)
0 (0%) (1e+00)
Layer6
C1
C2
C3
C4
C5
C6
0
1
2
3
4
- log10 (adjusted p-value)
