# Supplementary material for: Disruption of an Evolutionarily Novel Synaptic Expression Pattern in Autism
Source: PLoS Biol. 2016 Sep 29;14(9):e1002558. doi: 10.1371/journal.pbio.1002558 (PMC5042529; doi:10.1371/journal.pbio.1002558)
Supplement: S1 Table — (PDF) [file pbio.1002558.s026.pdf]

Species	Diagnosis	Sample ID	Year	Day	Gender	RIN*	Cause of death^	Source#	Random Order	Lane	Used[&]
Human	Autism	6399	2	0	M	7.3	Drowning	HBTRC	136	L7	1,3
Human	Autism	4671	4	165	F	8.7	Multiple injuries	NICHD-BTB	67	L4	1,3
Human	Autism	7002	5	0	F	8.4	Drowning	HBTRC	48	L3	1
Human	Autism	5569	5	0	M	5.3	Drowning	HBTRC	62	L3	1,2
Human	Autism	1349	5	220	M	7.4	Drowning	NICHD-BTB	120	L6	1
Human	Autism	5144	7	55	M	8.6	Cancer	NICHD-BTB	22	L1	1
Human	Autism	1174	7	283	F	5.4	Seizure disorder	NICHD-BTB	82	L4	
Human	Autism	5666	8	0	M	8.2	Sarcoma	HBTRC	149	L7	1,2
Human	Autism	4721	8	304	M	5.2	Drowning	NICHD-BTB	139	L7	1,2,3
Human	Autism	4925	9	0	M	7.6	Seizure disorder	HBTRC	84	L4	
Human	Autism	7741	10	0	M	8.3	Epilepsy	HBTRC	2	L1	1
Human	Autism	6831	10	0	F	8.3	Drowning	HBTRC	72	L4	1
Human	Autism	5342	11	0	F	6.5	Drowning	HBTRC	60	L3	1
Human	Autism	7078	11	0	M	5.5	Sudden unexpected death	HBTRC	113	L6	
Human	Autism	4899	14	126	M	8.2	Drowning	NICHD-BTB	7	L1	1,3
Human	Autism	7575	15	0	M	7	Head trauma	HBTRC	100	L5	
Human	Autism	6184	18	0	F	8	Seizure disorder	HBTRC	44	L2	1,2
Human	Autism	7718	19	0	M	6.7	Stopped breathing	HBTRC	96	L5	1
Human	Autism	5144h	20	0	M	8.1	Accident, auto trauma	HBTRC	109	L6	1,2,3
Human	Autism	1638	20	277	F	5.4	Seizure disorder	NICHD-BTB	122	L6	1
Human	Autism	6337	22	0	M	5.6	Aspiration	HBTRC	29	L2	1,3
Human	Autism	7769	24	0	M	8.2	ARDS	HBTRC	71	L4	1
Human	Autism	5000	27	0	M	4.2	Drowning	HBTRC	148	L7	
Human	Autism	6994	29	0	M	7.8	Seizure suspected	HBTRC	57	L3	1,2
Human	Autism	6640	29	0	F	6.3	Seizure disorder	HBTRC	116	L6	1,3
Human	Autism	5173	30	0	M	5.9	Gastrointestinal bleeding	HBTRC	53	L3	
Human	Autism	7762	30	0	M	7.4	Epilepsy	HBTRC	74	L4	
Human	Autism	6677	30	0	M	7.8	Congestive heart failure	HBTRC	134	L7	1,2
Human	Autism	6401	39	0	M	6.8	Cardiac tamponade	HBTRC	26	L2	1,2
Human	Autism	7672	39	0	M	2.6	Aspiration pneumonitis	HBTRC	144	L7	1
Human	Autism	7085	49	0	F	5.8	Colorectal cancer	HBTRC	73	L4	
Human	Autism	7886	50	0	M	7.6	Unknown	HBTRC	65	L4	
Human	Autism	7376	52	0	F	6	Unknown	HBTRC	79	L4	1,2
Human	Autism	7090	60	0	M	6.3	Pancreatic cancer	HBTRC	117	L6	1,3
Human	Control	447	0	2	M	8	Complications of prematurity	NICHD-BTB	93	L5	1
Human	Control	779	0	5	M	8.8	Congenital heart defect	NICHD-BTB	54	L3	1
Human	Control	398	0	16	F	8.8	Unknown	NICHD-BTB	25	L2	1
Human	Control	1157	0	20	F	7.1	Pneumonia associated	NICHD-	107	L6	1

							with meconium aspiration	BTB			
Human	Control	759	0	35	M	7.9	Idiopathic pulmonary hemorrhage	NICHD-BTB	132	L7	1
Human	Control	1055	0	96	M	7.7	Bronchopneumania	NICHD-BTB	70	L4	1
Human	Control	5183	0	107	M	7.7	SIDS	NICHD-BTB	129	L7	1
Human	Control	1303	0	212	M	8	Unknown	NICHD-BTB	127	L6	1
Human	Control	1453	1	78	M	6.7	Asthma	NICHD-BTB	51	L3	1
Human	Control	814	1	123	M	7.6	Drowning	NICHD-BTB	68	L4	1
Human	Control	1275	2	57	F	5.5	Acute myocarditis	NICHD-BTB	27	L2	1
Human	Control	1791	2	286	F	8.2	Drowning	NICHD-BTB	125	L6	1,3
Human	Control	6736	4	0	F	6.8	Acute bronchopneumonia	HBTRC	92	L5	1
Human	Control	1185	4	258	M	8.6	Drowning	NICHD-BTB	111	L6	1,3
Human	Control	4907	4	274	F	8.1	Asthma	NICHD-BTB	9	L1	1,2
Human	Control	4898	7	272	M	6.4	Drowning	NICHD-BTB	76	L4	1
Human	Control	1860	8	2	M	8	Cardiac arrhythmia	NICHD-BTB	36	L2	1,2,3
Human	Control	1706	8	214	F	7.1	Rejection of cardiac allograft transplantation	NICHD-BTB	137	L7	1
Human	Control	5161	10	262	F	7.4	Hanging	NICHD-BTB	14	L1	1
Human	Control	M3228	11	294	M	8.3	Internal bleeding	NICHD-BTB	110	L6	1
Human	Control	1908	13	360	M	8.2	Hanging	NICHD-BTB	87	L5	1,3
Human	Control	M3830	14	250	M	6.1	Hanging	NICHD-BTB	80	L4	
Human	Control	5242	15	119	M	8.4	Cardiac arrythmia	NICHD-BTB	143	L7	1
Human	Control	7387	17	0	M	8.1	Hanging	HBTRC	98	L5	1,2
Human	Control	5251	19	0	M	6.6	Respiratory infection	HBTRC	49	L3	1
Human	Control	4548	20	63	F	8.7	Multiple injures	NICHD-BTB	141	L7	1,2,3
Human	Control	1846	20	221	F	7.1	Multiple injures	NICHD-BTB	42	L2	1
Human	Control	1442	22	322	M	7.5	Multiple injures	NICHD-BTB	99	L5	1,3
Human	Control	7738	24	0	M	7.2	Unknown	HBTRC	37	L2	1
Human	Control	602	27	42	M	8.8	Multiple injuries	NICHD-BTB	59	L3	1
Human	Control	B-24	28	0	F	6.5	Unknown	MPRC	130	L7	1,2,3
Human	Control	1502	29	363	M	7.4	Multiple injuries	NICHD-BTB	77	L4	1,2

Human	Control	7344	36	0	F	6.6	Multisystem failure	HBTRC	45	L3	1
Human	Control	7369	36	0	M	5.7	Possible pulmonary embolism	HBTRC	50	L3	
Human	Control	7561	39	0	M	7	Heart attack	HBTRC	64	L3	1,2
Human	Control	1134	41	241	M	8.9	ASCVD	NICHD-BTB	97	L5	1
Human	Control	6259	50	0	M	8	Unknown	HBTRC	133	L7	1,2
Human	Control	1578	53	112	M	8.3	ASCVD	NICHD-BTB	21	L1	1
Human	Control	6860	56	0	M	8	Unknown	HBTRC	81	L4	1
Human	Control	4263	61	187	M	8.8	Cardiac arrest	NICHD-BTB	23	L2	1,3
Chimpanzee	Control	11454	0	0	M	6.9	-	BZ	138	L7	1
Chimpanzee	Control	13302	0	1	F	7.5	-	BZ	118	L6	1
Chimpanzee	Control	13308	0	7	M	2.3	-	BZ	17	L1	
Chimpanzee	Control	58	0	7	F	8.2	-	SNPRC	20	L1	1
Chimpanzee	Control	13311	0	8	M	7.9	-	BZ	63	L3	1
Chimpanzee	Control	60	0	32	F	7.3	-	SNPRC	18	L1	1
Chimpanzee	Control	59	0	34	M	5.8	-	SNPRC	112	L6	1
Chimpanzee	Control	13309	0	39	M	5.6	-	BZ	35	L2	
Chimpanzee	Control	13304	0	45	F	6.6	-	BZ	24	L2	1
Chimpanzee	Control	11452	1	160	F	7.9	-	BZ	106	L5	1
Chimpanzee	Control	13312	6	123	F	5.7	-	BZ	104	L5	
Chimpanzee	Control	57	10	146	M	6.6	-	SNPRC	147	L7	1
Chimpanzee	Control	Japie	12	35	M	6.9	-	BPRC	39	L2	
Chimpanzee	Control	37	16	219	F	5.9	-	SNPRC	124	L6	
Chimpanzee	Control	38	16	292	F	3.4	-	SNPRC	30	L2	
Chimpanzee	Control	1480	17	292	F	6.7	-	GW	16	L1	1
Chimpanzee	Control	40	18	0	F	7.8	-	SNPRC	40	L2	1
Chimpanzee	Control	CAO127	18	183	F	7.3	-	GW	11	L1	1
Chimpanzee	Control	31	20	73	F	8	-	SNPRC	56	L3	1
Chimpanzee	Control	32	20	183	F	5.8	-	SNPRC	85	L4	1
Chimpanzee	Control	30	21	110	M	5.6	-	SNPRC	126	L6	1
Chimpanzee	Control	1465	21	146	F	6.1	-	GW	8	L1	1
Chimpanzee	Control	1437	21	183	F	7.1	-	GW	46	L3	1
Chimpanzee	Control	1275	22	256	M	7.7	-	GW	66	L4	1
Chimpanzee	Control	1365	23	329	M	8.2	-	GW	38	L2	
Chimpanzee	Control	1358	24	37	M	7.7	-	GW	90	L5	1
Chimpanzee	Control	1028	25	183	M	2.4	-	GW	119	L6	
Chimpanzee	Control	1351	26	146	M	8.2	-	GW	34	L2	1
Chimpanzee	Control	1266	27	37	M	8.1	-	GW	75	L4	1
Chimpanzee	Control	1342	28	329	M	5.7	-	GW	94	L5	1
Chimpanzee	Control	1167	29	0	M	7.9	-	GW	108	L6	1
Chimpanzee	Control	936	30	292	F	5.7	-	GW	103	L5	1
Chimpanzee	Control	1060	31	73	M	7.5	-	GW	102	L5	1
Chimpanzee	Control	53	32	146	M	5.8	-	SNPRC	91	L5	1
Chimpanzee	Control	905	34	183	M	7.6	-	GW	43	L2	1
Chimpanzee	Control	Zurich	35	9	M	5.8	-	BZ	105	L5	1
Chimpanzee	Control	5	39	329	F	8	-	SNPRC	89	L5	1
Chimpanzee	Control	1045	42	219	M	7.7	-	GW	146	L7	1

Chimpanzee	Control	1560	109	329	M	6.7	-	GW	12	L1	1
Macaque	Control	NB0903	0	0.5	M	8.6	-	Suzhou	145	L7	1
Macaque	Control	NB0901	0	1	M	8.5	-	Suzhou	10	L1	1
Macaque	Control	NB0905	0	7	M	9.3	-	Suzhou	5	L1	1
Macaque	Control	705	0	16	M	9.1	-	Suzhou	61	L3	1
Macaque	Control	704	0	20	M	8.4	-	Suzhou	19	L1	1
Macaque	Control	702	0	23	M	9.5	-	Suzhou	114	L6	1
Macaque	Control	701	0	24	M	9	-	Suzhou	55	L3	
Macaque	Control	70175	0	151	M	9.6	-	Suzhou	131	L7	1
Macaque	Control	70115	0	179	M	8.5	-	Suzhou	128	L6	1
Macaque	Control	70133	0	207	M	8.3	-	Suzhou	47	L3	1
Macaque	Control	6709	0	278	M	8.7	-	Suzhou	83	L4	1
Macaque	Control	6237	0	353	M	9	-	Suzhou	123	L6	1
Macaque	Control	61569	1	80	M	8.3	-	Suzhou	142	L7	1
Macaque	Control	51087	1	170	M	9.3	-	Suzhou	140	L7	
Macaque	Control	51373	1	242	M	9.1	-	Suzhou	52	L3	1
Macaque	Control	51469	1	294	M	8.1	-	Suzhou	78	L4	1
Macaque	Control	51095	2	9	M	9	-	Suzhou	4	L1	
Macaque	Control	5773	2	101	M	8.4	-	Suzhou	69	L4	1
Macaque	Control	4093	3	40	M	9.3	-	Suzhou	135	L7	1
Macaque	Control	50715	3	80	M	8.7	-	Suzhou	6	L1	1
Macaque	Control	4089	3	110	M	8.9	-	Suzhou	121	L6	1
Macaque	Control	3071	4	27	M	7.8	-	Suzhou	101	L5	1
Macaque	Control	B00051	6	165	M	8.2	-	Suzhou	58	L3	1
Macaque	Control	135	7	15	M	8.7	-	Suzhou	115	L6	1
Macaque	Control	99057	8	16	M	9.3	-	Suzhou	33	L2	
Macaque	Control	98145	9	37	M	7.8	-	Suzhou	1	L1	
Macaque	Control	98073	9	104	M	8.5	-	Suzhou	15	L1	1
Macaque	Control	96007	10	328	M	8.4	-	Suzhou	28	L2	1
Macaque	Control	95001	11	346	M	8.4	-	Suzhou	95	L5	1
Macaque	Control	94051	13	17	M	8.7	-	Suzhou	32	L2	1
Macaque	Control	93041	14	21	M	8.9	-	Suzhou	41	L2	1
Macaque	Control	92095	14	349	M	8.2	-	Suzhou	88	L5	1
Macaque	Control	92107	15	3	M	7.8	-	Suzhou	86	L5	1
Macaque	Control	90049	17	22	M	9	-	Suzhou	3	L1	1
Macaque	Control	87015	20	91	M	7.9	-	Suzhou	31	L2	1
Macaque	Control	86023	21	8	M	5.9	-	Suzhou	13	L1	1

* RIN: RNA Integrity Number;

^ ARDS: Acute respiratory distress syndrome;

ASCVD: Arteriosclerotic cardiovascular disease;

SIDS: Sudden infant death syndrome.

NICHD-BTB: NICHD Brain and Tissue Bank for Developmental Disorders at the University of Maryland;

HBTRC: Harvard Brain Tissue Resource Center;

MPRC: Maryland Brain Collection Center;

BPRC: Biomedical Primate Research Centre;

BZ: Burgers' Zoo in Arnhem;

SNPRC: Southwest National Primate Research Center, San Antonio, TX;

GW: Department of Anthropology, The George Washington University, Washington, DC;

Suzhou: Suzhou Drug Safety Evaluation and Research Center, Suzhou, China.

& 1: samples with good quality used in the main analysis;

2: age-matched samples used to estimate the expression change in autism between RNA-seq and array;

3: age-matched samples used to estimate the expression change in autism between RNA-seq and ChIP-seq.
