# Supplementary material for: Disruption of an Evolutionarily Novel Synaptic Expression Pattern in Autism
Source: PLoS Biol. 2016 Sep 29;14(9):e1002558. doi: 10.1371/journal.pbio.1002558 (PMC5042529; doi:10.1371/journal.pbio.1002558)
Supplement: S2 Table — (PDF) [file pbio.1002558.s027.pdf]

Species	Diagnosis	Sample ID	Total reads	Mapped reads		Unique mapped	
				genome	junction	genome	junction
Human	Autism	6399	10341810	7740346	1691148	7224893	1536161
Human	Autism	4671	11251222	8442004	1798371	7670246	1634228
Human	Autism	5569	9365139	7479459	1153000	6912609	1031658
Human	Autism	7002	10129699	7469097	1727917	7056950	1581828
Human	Autism	1349	11576594	9050799	1638023	8150124	1452079
Human	Autism	5144	5650860	4924661	230423	1580469	210376
Human	Autism	1174	9821150	8221332	664805	7114705	533988
Human	Autism	5666	10567561	7998771	1716611	7549617	1547295
Human	Autism	4721	9404085	7429888	1225921	6369888	1096009
Human	Autism	4925	9581455	7555085	1227527	7006753	1084434
Human	Autism	6831	9505124	7079304	1565018	6425471	1434347
Human	Autism	7741	10093305	7842256	1506187	7349180	1325104
Human	Autism	5342	8131743	6803651	718898	6145559	620328
Human	Autism	7078	8532516	6794499	1124431	6304172	939922
Human	Autism	4899	9039432	6953140	1402557	6255257	1248693
Human	Autism	7575	7155438	5106535	1504441	4599477	1190560
Human	Autism	6184	8502885	6405799	1415526	5976095	1291123
Human	Autism	7718	8858576	6757707	1349585	6352885	1201700
Human	Autism	5144h	11519086	8721028	1765189	7594697	1604139
Human	Autism	1638	10726057	8546126	1360062	7581203	1230738
Human	Autism	6337	7847515	6717720	509170	5690843	445834
Human	Autism	7769	9211131	7115073	1409689	6656306	1230538
Human	Autism	5000	10929117	8397675	1669412	7642242	1421034
Human	Autism	6640	9567591	7359513	1555961	6456256	1343032
Human	Autism	6994	8898070	6913235	1358148	6411937	1220633
Human	Autism	5173	10198139	8552188	908293	7816192	794168
Human	Autism	6677	10179488	7947918	1388003	7374699	1246594
Human	Autism	7762	8076204	6515579	917675	6019828	802137
Human	Autism	6401	9448086	7054494	1608074	6559532	1417017
Human	Autism	7672	7923118	7033762	322770	6281152	263674
Human	Autism	7085	10662256	8700074	1116787	7926517	953812
Human	Autism	7886	8171231	6421488	1053504	5745544	895261
Human	Autism	7376	10811206	8306460	1681903	7732890	1506186
Human	Autism	7090	8770747	7027406	1037721	6286034	905311
Human	Control	447	10904288	4489372	962356	4009137	812180
Human	Control	779	10466812	7878598	1713179	7204652	1510454
Human	Control	398	7919215	5689593	1753556	5128979	1430046
Human	Control	1157	9303221	7251098	1424648	6558155	1169327
Human	Control	759	9657862	6725619	2070676	6144772	1910054
Human	Control	1055	11282445	8968968	1319992	8195650	1144021
Human	Control	5183	11044497	8844191	1349604	8181322	1202368
Human	Control	1303	11070436	8714358	1404567	7766008	1257876
Human	Control	1453	10544783	7870411	1556445	7358725	1414755
Human	Control	814	10476563	7774084	1804458	6757047	1624708
Human	Control	1275	9734021	7519592	1454762	7055552	1319010
Human	Control	1791	12127944	9234272	1957074	8378456	1775492

Human	Control	6736	8326903	6602524	1110138	6150559	990938
Human	Control	1185	9786434	7227661	1766503	6012267	1598969
Human	Control	4907	9578007	7381572	1431162	6633364	1280551
Human	Control	4898	11760445	8870468	1849291	8247486	1671575
Human	Control	1860	8670080	6644239	1408204	5670310	1274383
Human	Control	1706	11905741	9524505	1372170	8028633	1237169
Human	Control	5161	8367710	6482585	1270175	6032934	1142569
Human	Control	M3228	11329437	8336202	1971448	7894501	1813212
Human	Control	1908	9433644	7213477	1499775	6630406	1359520
Human	Control	M3830	10528550	8116461	1532680	7616449	1392696
Human	Control	5242	11234835	8258652	1984180	7786160	_1862765
Human	Control	7387	9126544	6760065	1583226	6370675	1457380
Human	Control	5251	9723637	7802368	1214133	7211860	1084998
Human	Control	4548	11156736	8077140	2075023	7246965	1897383
Human	Control	1846	7406407	5593446	1196004	5158518	1055831
Human	Control	1442	9292950	6943472	1426547	5630543	1302083
Human	Control	7738	7769759	6216512	1032869	5769998	910867
Human	Control	602	9035360	6814642	1533618	5900515	1361529
Human	Control	B-24	11081762	8376822	1880885	7768838	1692326
Human	Control	1502	10013837	7466882	1804950	6812034	1624793
Human	Control	7344	9659056	7418503	1499796	6885165	1331794
Human	Control	7369	10213241	8488212	460217	7630620	401695
Human	Control	7561	10017170	7704910	1510348	7234303	1350277
Human	Control	1134	8998020	6659671	1488452	5214083	1358892
Human	Control	6259	11166137	8516050	1800989	8009260	1630728
Human	Control	1578	10028528	7422955	1720335	6766947	1539136
Human	Control	6860	10726199	7963374	1680476	7484192	1524838
Human	Control	4263	8467944	6585842	1241794	5726512	1079641
Chimpanzee	Control	11454	8827503	6596771	1203050	6128632	1022657
Chimpanzee	Control	13302	10521488	7681022	1333456	7069382	1151304
Chimpanzee	Control	58	9989855	7165479	1516320	6768922	1336570
Chimpanzee	Control	13308	8976485	6938033	716199	6346687	578483
Chimpanzee	Control	13311	10713100	7724901	1375759	7180469	1222709
Chimpanzee	Control	60	6987426	4974941	949371	4656329	800106
Chimpanzee	Control	59	11340002	8274524	1455468	7679805	1238474
Chimpanzee	Control	13309	8351209	6250212	752427	5772295	621901
Chimpanzee	Control	13304	8029644	5897778	926518	5524941	806275
Chimpanzee	Control	11452	9281689	6738744	1181803	6298632	1075244
Chimpanzee	Control	13312	8597128	6256326	586011	5517749	506698
Chimpanzee	Control	57	11226560	7979186	1527518	7424635	1379665
Chimpanzee	Control	1560	8717372	6365697	1288698	5923136	1149432
Chimpanzee	Control	Japie	9231851	7017852	1140523	6623278	999590
Chimpanzee	Control	37	8792020	6323232	1044433	5889417	855595
Chimpanzee	Control	38	8185238	5975570	867752	5494188	739112
Chimpanzee	Control	1480	13079392	9401497	1624595	8837360	1475072
Chimpanzee	Control	40	8501118	6112583	1106923	5716417	984470
Chimpanzee	Control	CAO127	9747361	7149339	1401638	6688306	1251974
Chimpanzee	Control	31	9897509	7058897	1461751	6656261	1323243
Chimpanzee	Control	32	10754703	7800575	1483971	7326169	1350811

Chimpanzee	Control	30	10727964	7914321	1525708	7410966	1316314
Chimpanzee	Control	1465	10385964	7381675	1405031	6937196	1249635
Chimpanzee	Control	1437	10144166	7639440	1358308	7225426	1212525
Chimpanzee	Control	1275	11599628	8392849	1731709	7971912	1577050
Chimpanzee	Control	1365	8931801	6561757	1248897	6248975	1137883
Chimpanzee	Control	1358	8961884	6489079	1355879	6054623	1199204
Chimpanzee	Control	1028	8509062	6422346	805448	5855359	640075
Chimpanzee	Control	1351	8686063	6092013	1308341	5726189	1193715
Chimpanzee	Control	1266	12190487	8645727	1806353	8149299	1644607
Chimpanzee	Control	1342	8814780	6597796	1109744	6142663	1004399
Chimpanzee	Control	1167	11260122	8110292	1765337	7691259	1607212
Chimpanzee	Control	936	7975301	5958703	1019419	5562706	914032
Chimpanzee	Control	1060	8035483	5598345	1182438	5134670	1028498
Chimpanzee	Control	53	11244844	8209323	1623368	7707904	1470897
Chimpanzee	Control	905	8426240	6020879	1047985	5488925	926909
Chimpanzee	Control	Zurich	9344238	6658947	1085971	6123666	973671
Chimpanzee	Control	5	9675462	6972505	1448451	6570756	1325423
Chimpanzee	Control	1045	9709081	6961605	1252612	6525393	1114358
Macaque	Control	NB0903	11502454	7443519	1791433	7072398	1627879
Macaque	Control	NB0901	12245658	7957389	1828244	7589562	1656837
Macaque	Control	NB0905	11759873	7552310	1818038	7203181	1660040
Macaque	Control	0705	10161033	6601120	1494305	6272905	1361025
Macaque	Control	704	11744435	7597369	1667284	7206602	1510995
Macaque	Control	0702	12066253	7771658	1891508	7404647	1725309
Macaque	Control	0701	10161313	6145894	1687432	5797959	1531189
Macaque	Control	070175	10828433	6934072	1634044	6587060	1488922
Macaque	Control	70115	8370651	5281263	1187885	5029208	1082139
Macaque	Control	70133	9509000	6088097	1379218	5779705	1253349
Macaque	Control	6709	12644449	8182577	2006667	7816430	1831113
Macaque	Control	06237	13122198	8516379	1884174	8083893	1708261
Macaque	Control	61569	10515498	6872019	1587885	6543079	1436963
Macaque	Control	051087	10612063	6579844	1773421	6196291	1591120
Macaque	Control	051373	11516693	7303719	1736128	6946186	1585947
Macaque	Control	051469	12620439	8144785	1812148	7729112	1632282
Macaque	Control	051095	10806008	6382176	1851286	5934626	1658036
Macaque	Control	05773	10881989	6972735	1623767	6557560	1448623
Macaque	Control	4093	12402852	7889153	1897576	7488790	1741383
Macaque	Control	050715	9546532	6165231	1512163	5881092	1372731
Macaque	Control	04089	13305974	8399817	2083689	8007029	1907169
Macaque	Control	3071	9474277	5919447	1437051	5618115	1313355
Macaque	Control	B00051	10268844	6438830	1427277	6058721	1299720
Macaque	Control	00135	12394922	7834746	1906518	7425358	1721112
Macaque	Control	99057	9004028	5351051	1544764	5015488	1395599
Macaque	Control	98145	11156709	6795232	1798172	6354846	1591960
Macaque	Control	98073	18078522	10345918	2271077	9666772	2041925
Macaque	Control	96007	10598207	6752562	1659475	6431279	1510588
Macaque	Control	95001	10326175	6321662	1549415	6002785	1409098
Macaque	Control	94051	9309516	5887079	1389752	5581685	1264320
Macaque	Control	93041	9886005	6224126	1460464	5879772	1333081

Macaque	Control	92095	9514593	6079328	1392926	5756198	1259834
Macaque	Control	92107	10228429	6514166	1446672	6151052	1303298
Macaque	Control	90049	13407744	8637289	2110619	8218700	1905997
Macaque	Control	87015	9325483	6046863	1364216	5683741	1204434
Macaque	Control	86023	11968615	7823376	1763733	7402794	1579585
