# Supplementary material for: Disruption of an Evolutionarily Novel Synaptic Expression Pattern in Autism
Source: PLoS Biol. 2016 Sep 29;14(9):e1002558. doi: 10.1371/journal.pbio.1002558 (PMC5042529; doi:10.1371/journal.pbio.1002558)
Supplement: S3 Table — (PDF) [file pbio.1002558.s028.pdf]

Cluster		Function annotation	Observed	Con trol	Adjust p-value
Cluster 1	KEGG	Ribosome	37	1	7.24E-17
		Valine, leucine and isoleucine degradation	7	0	2.10E-03
		Fatty acid metabolism	9	1	2.10E-03
	GO	Transcription from RNA polymerase II promoter	41	42	2.30E-02
		Translation	55	33	2.41E-07
		Gene expression	163	245	2.68E-03
		Positive regulation of transcription from RNA polymerase II promoter	16	10	3.78E-02
		Positive regulation of transcription	27	23	3.12E-02
		Cellular metabolic process	279	503	3.12E-02
		Biosynthetic process	194	276	1.59E-05
		Translational elongation	39	2	2.55E-15
		Positive regulation of transcription, DNA-dependent	22	15	2.21E-02
		Response to lipopolysaccharide	6	0	3.12E-02
		Negative regulation of biosynthetic process	28	25	3.18E-02
		Cellular biosynthetic process	194	270	5.65E-06
		Positive regulation of gene expression	27	24	3.20E-02
		Positive regulation of cellular biosynthetic process	32	31	3.19E-02
		Carboxylic acid catabolic process	13	7	4.84E-02
		Macromolecule biosynthetic process	160	214	1.92E-05
	Structure	White matter	65	31	5.17E-32
	Cell type	Astrocytes	149	67	9.86E-23
Cluster 2	KEGG	Calcium signaling pathway	16	7	2.11E-05
		Dilated cardiomyopathy	7	1	1.51E-03
		Long-term potentiation	7	5	4.33E-02
	GO	Homophilic cell adhesion	16	4	1.26E-04
		Cell adhesion	38	59	1.91E-02
		Calcium ion transport	18	10	4.69E-04
		Protein polymerization	8	1	2.92E-03
		Synaptic transmission	25	19	4.04E-04
		Transport	117	238	3.75E-03
		Cell-cell signaling	32	31	4.04E-04
		Nervous system development	50	80	7.14E-03
		Cell-cell adhesion	21	14	4.69E-04
		Actin filament polymerization	7	1	6.71E-03
		Cell communication	123	267	1.47E-02
		Metal ion transport	36	33	1.61E-04
		Ion transport	46	57	4.04E-04
		Potassium ion transport	14	12	2.00E-02
		Regulation of actin cytoskeleton organization	7	1	6.71E-03
		Cation transport	39	41	3.51E-04

		Sodium ion transport	13	7	3.75E-03
		Regulation of synaptic transmission	9	4	1.47E-02
		Monovalent inorganic cation transport	24	23	2.92E-03
		Transmission of nerve impulse	27	22	4.04E-04
		Actin filament organization	9	5	2.65E-02
		Sphingolipid metabolic process	6	2	4.70E-02
		Cellular protein complex assembly	15	12	1.11E-02
		Actin polymerization or depolymerization	8	1	2.92E-03
		Biological adhesion	38	59	1.91E-02
		Membrane lipid metabolic process	6	2	4.70E-02
		Establishment of localization	117	238	3.75E-03
		Di-, tri-valent inorganic cation transport	19	15	2.99E-03
	Structure	Grey matter	136	96	3.95E-27
	Cell type	Pnoc+ Neurons	32	65	2.01E-05
		Layer 5b Neurons	27	29	2.20E-08
Cluster 3	KEGG	Huntington's disease	8	12	3.50E-02
		Aminoacyl-tRNA biosynthesis	7	3	1.85E-03
		Parkinson's disease	6	1	1.60E-03
		Glycolysis / Gluconeogenesis	7	5	5.61E-03
	GO	Vesicle-mediated transport	30	49	6.21E-04
		Transport	86	269	4.56E-03
		Cellular macromolecule catabolic process	24	54	3.53E-02
		Glucose metabolic process	12	9	1.85E-03
		Aerobic respiration	6	1	4.56E-03
		Glycolysis	11	4	3.55E-04
		Generation of precursor metabolites and energy	24	29	3.55E-04
		Cellular respiration	9	7	9.04E-03
		Intracellular transport	32	83	4.16E-02
		Membrane organization	18	26	4.69E-03
		Catabolic process	46	122	9.04E-03
		Dicarboxylic acid metabolic process	6	2	9.33E-03
		Alcohol catabolic process	12	8	1.13E-03
		Establishment of localization in cell	39	100	1.33E-02
		Glucose catabolic process	11	6	1.13E-03
		Hexose metabolic process	13	14	4.97E-03
		Monosaccharide metabolic process	14	17	6.07E-03
		Establishment of localization	86	269	4.56E-03
	Structure	Grey matter	73	159	5.18E-12
	Cell type	Pnoc+ Neurons	23	74	7.30E-03
		Layer 5b Neurons	25	31	2.10E-08
		Layer 5a Neurons	14	18	7.16E-05
Cluster 4	GO	RNA splicing	13	20	3.31E-02
		Immune response	19	37	2.84E-02
		mRNA processing	16	23	1.88E-02
		Nucleobase, nucleoside, nucleotide and nucleic acid metabolic process	91	331	2.84E-02
		Defense response	19	37	2.84E-02

		mRNA metabolic process	20	24	1.01E-03
		RNA metabolic process	59	198	4.60E-02
		RNA processing	24	48	1.88E-02
		Nitrogen compound metabolic process	95	362	4.80E-02
		Positive regulation of immune system process	9	11	4.60E-02
		Regulation of T cell activation	6	4	4.60E-02
		Immune system process	26	60	2.84E-02
	Structure	White matter	25	71	7.95E-13
Cluster 5	GO	Regulation of transcription	17	226	1.50E-02
		Transcription	17	243	2.06E-02
		Nucleobase, nucleoside, nucleotide and nucleic acid metabolic process	21	401	4.80E-02
		Biosynthetic process	23	447	4.14E-02
		Regulation of gene expression	17	256	2.36E-02
		Regulation of metabolic process	19	317	3.17E-02
		Negative regulation of biosynthetic process	6	47	3.37E-02
		Cellular biosynthetic process	23	441	3.87E-02
		Regulation of cellular metabolic process	19	295	2.06E-02
		Regulation of nitrogen compound metabolic process	18	242	1.50E-02
		Macromolecule biosynthetic process	20	354	3.52E-02
	Structure	Grey matter	9	223	4.23E-02
	Cell type	CCK-expressing neurons	8	33	8.04E-04
Cluster 6	GO	Regulation of cellular process	25	610	1.55E-02
	Cell type	Oligodendrocytes	8	39	9.73E-06
