# Supplementary material for: Disruption of an Evolutionarily Novel Synaptic Expression Pattern in Autism
Source: PLoS Biol. 2016 Sep 29;14(9):e1002558. doi: 10.1371/journal.pbio.1002558 (PMC5042529; doi:10.1371/journal.pbio.1002558)
Supplement: S4 Table — (PDF) [file pbio.1002558.s029.pdf]

Sample ID	ADI-R domain scores*				Groups**
	Social interaction	Stereotyped pattern	Development evident	Sum	
6640	30	12	5	47	high 1
6677	26	12	5	43	high 2
6184	28	9	5	42	high 2
6337	28	6	5	39	high 2
5144h	27	6	4	37	moderate 1
4721	24	10	3	37	moderate 1
7090	26	4	5	35	moderate 1
7002	24	7	4	35	moderate 1
5144	28	3	3	34	moderate 1
4671	26	3	5	34	moderate 1
4899	22	8	4	34	moderate 1
4925	24	4	5	33	moderate 1
5569	22	6	5	33	moderate 1
1174	22	6	5	33	moderate 1
1638	21	7	5	33	moderate 1
7078	22	4	5	31	moderate 2
6994	22	5	3	30	moderate 2
5342	22	3	5	30	moderate 2
5173	22	2	5	29	moderate 2
6401	20	4	5	29	moderate 2
5666	19	4	2	25	low
6399	14	6	5	25	low

*ADI-R domain scores: Autism Diagnostic Interview Communication score.

**Autistic individuals were grouped by the sum of the ADI-R domain scores.
