# Supplementary material for: Disruption of an Evolutionarily Novel Synaptic Expression Pattern in Autism
Source: PLoS Biol. 2016 Sep 29;14(9):e1002558. doi: 10.1371/journal.pbio.1002558 (PMC5042529; doi:10.1371/journal.pbio.1002558)
Supplement: S5 Table — (PDF) [file pbio.1002558.s030.pdf]

	Function annotation	Observed	Control	Adjust p-value
KEGG	Calcium signaling pathway	6	113	1.29E-03
	MAPK signaling pathway	7	200	2.34E-03
	Homophilic cell adhesion	6	87	3.25E-03
	Calcium ion transport	9	148	9.88E-04
	Synaptic transmission	9	282	9.94E-03
	Cell-cell signaling	10	433	3.89E-02
	Protein amino acid dephosphorylation	6	110	6.47E-03
	Dephosphorylation	7	144	4.83E-03
	Cell communication	39	2864	1.35E-02
	Metal ion transport	14	441	1.39E-03
	Ion transport	16	645	3.25E-03
	Cation transport	14	507	3.25E-03
	Sodium ion transport	6	98	4.63E-03
	Transmission of nerve impulse	10	326	7.64E-03
	Cellular homeostasis	9	320	1.96E-02
	Regulation of cell communication	15	756	2.13E-02
GO	Di-, trivalent inorganic cation transport	9	177	1.39E-03
