# Supplementary material for: Disruption of an Evolutionarily Novel Synaptic Expression Pattern in Autism
Source: PLoS Biol. 2016 Sep 29;14(9):e1002558. doi: 10.1371/journal.pbio.1002558 (PMC5042529; doi:10.1371/journal.pbio.1002558)
Supplement: S6 Table — (PDF) [file pbio.1002558.s031.pdf]

Targets	EGR member	Number*	Source	Cell lines	GEO Number	Reference
predict	EGR1-4	852	Match algorithm	-	-	-
egr1_Ecc1	EGR1	3237	ChIP-Seq	ECC-1	GSM1010888	Pope et al, 2014, Nature
egr1_Hct116	EGR1	2658	ChIP-Seq	HCT-116	GSM1010846	Pope et al, 2014, Nature
egr1_Mcf7	EGR1	3034	ChIP-Seq	MCF-7	GSM1010844	Pope et al, 2014, Nature
egr1_Gm12878	EGR1	6373	ChIP-Seq	GM12878	GSM803434	Pope et al, 2014, Nature
egr1_H1hesc	EGR1	3783	ChIP-Seq	H1-hESC	GSM803430	Pope et al, 2014, Nature
egr1_K562	EGR1	8298	ChIP-Seq	K562	GSM803414	Pope et al, 2014, Nature
egr2_MAK	EGR2	4970	ChIP-Seq	MAC#	GSM785503, GSM785504	Pham et al, 2012, Blood
egr1_LoVo	EGR1	3511	ChIP-Seq	LoVo	GSM1208608	Yan et al, 2013, Cell

*: Only target genes detected as expressed in our human brain data are shown.

#: Monocyte-derived macrophage
