# Supplementary material for: Disruption of an Evolutionarily Novel Synaptic Expression Pattern in Autism
Source: PLoS Biol. 2016 Sep 29;14(9):e1002558. doi: 10.1371/journal.pbio.1002558 (PMC5042529; doi:10.1371/journal.pbio.1002558)
Supplement: S1 Text — (PDF) [file pbio.1002558.s032.pdf]

Supplementary Methods

RNA-seq data quality and variance estimation

We estimated the data quality of each sample using an estimate of the 5'/3' bias of the sequence coverage as a proxy for RNA degradation. We scanned the reads distribution across gene body using "geneBody_coverage" module in the RSeQC package (Wang et al., 2012). Samples with regression coefficient of the reads distribution curve greater than 0.013 were removed from further analysis. Outliers were further removed based on principle components analysis of the expression data performed using the "prcomp" function in the R "stats" package. In total, 38 control, 25 autism, 31 chimpanzee and 31 macaque samples were retained in downstream analysis. The RNA quality estimated using "geneBody_coverage" module correlated well with the RNA integrity values (RIN) measured by Agilent Bioanalyzer (Pearson correlation, $r = -0.62$, $p < 0.001$).

We used Principal Variance Component Analysis (PVCA) to calculate the variance explained by various factors among human samples as described elsewhere (Boedigheimer et al., 2008; Fu et al., 2011; Li et al., 2009). In brief, principal components explaining more than 5% the total variance were used as response variables to fit a mixed linear model with different sources of variation, such as disease status, age, sex, RNA quality estimated using "geneBody_coverage" module as described above, and experimental batch information as random effects, using "lmer" function in the R package "lme4". The model was fit using restricted maximum likelihood (REML) to get the variance component estimates. The weighted average variance was then calculated based on the eigenvalues obtained in the principal component analysis.

Identifying the differentially expressed genes in autism by ANCOVA

We identified differential expression between autistic samples and healthy controls using analysis of covariance (ANCOVA) (Faraway, 2002) as described in (Somel et al., 2009b). Briefly, the test aims to identify whether disease and control groups have different curves of age-dependent expression changes. The test is performed twice, using one of the two groups as a reference per time. For each gene in the reference group, the effect of age on expression level, by using RPKM values as inputs, is estimated using polynomial regression models: we choose the best polynomial regression with age as predictor and expression level as response from a family of polynomial regression models using the "adjusted r^2" criterion (Faraway, 2002). We then test whether such a

regression model with disease-status parameters is significantly better than the model with common parameters for both groups. For example, if the null model for gene *i* was a linear one:

$$Y_{ij} = \beta_{0i} + \beta_{1i}A_j + \varepsilon_{ij},$$

we compare it to the alternative model:

$$Y_{ij} = \beta_{0iC} + \beta_{0iA} + \beta_{1iC}A_{jC} + \beta_{1iA}A_{jA} + \varepsilon_{ij},$$

where Y_{ij} is the expression level of individual *j*, β_{0iC} and β_{1iC} are the control-specific intercept and slope, β_{0iA} and β_{1iA} are the autism-specific intercept and slope, A_{jC} and A_{jA} are control and autism ages, respectively. The null model (with no disease-status parameters) and alternative models are compared using the F-test. We test each sub-model of the full alternative model, each containing one or more disease- status parameters. For each gene, if either of the two tests was significant at a defined cutoff, we considered it as differentially expressed in autism. The permutations were performed by dividing samples into eight age intervals and permuting disease-status identifiers within each interval in order to preserve the age-structure of the data. The FDR was calculated by 1,000 random permutations of sample labels.

Normalization of expression levels of primates and autism microarray datasets

In order to directly compare age-dependent expression profiles between published autism data and non-human primate data, we normalized the expression profiles from a primates microarray dataset and an autism microarray dataset measured in two different studies (Liu et al., 2012; Voineagu et al., 2011) using the following procedure: (i) we quantile normalized the expression values of the genes across both datasets (gene number = 8,431); (ii) within each dataset we chose sample subsets consisting of five age-matched human controls with ages from 20 to 60 years; and (iii) we standardized the mean of each dataset based on the sample subsets: for each gene in each dataset, we subtracted the mean calculated using a subset of five individuals subsampled from this dataset. We then added the combined mean based on all 10 subsampled individuals to all genes in both datasets.

Comparison of expression change between autism and control in RNA-seq and array

To estimate the robustness of gene expression changes in autism detected in our data, we compared it to a published study conducted in PFC of individuals with autism and healthy controls using microarrays (Liu et al., 2012; Voineagu et al., 2011). The expression difference

was calculated based on 8 pairs of age-matched autism and control samples measured in both datasets (Figure S1). We then calculated the mean expression change between autism and control across these age-matched samples in each dataset. The comparison of mean expression changes in autism in two datasets was used to test the robustness of expression change estimates.

Comparison of expression change between autism and control at gene expression level and H3K4me3 modification level

H3K4me3 modification data from 16 autism samples and 14 age-matched controls conducted by ChIP-Seq was downloaded from (Shulha et al., 2011). The raw sequencing reads were mapped to human genome (hg19) by Bowtie (Langmead et al., 2009) allowing up to two mismatches. Only uniquely mapped reads were used in downstream analysis.

To calculate the correlation of gene expression and H3K4me3 histone modification changes in autism, we classified the 4 kb region surrounding the transcription start site (TSS), defined using Ensembl human gene annotation (v66), as a promoter region (Cheung et al., 2010). For each sample, number of reads within each promoter region was normalized by the total reads mapped to all promoter regions in a sample. The normalized read coverage was used to perform the correlation with gene expression measurements. Specifically, for each gene in the control or autism group, the expression difference or modification difference was calculated based on 8 pairs of age-matched autism and control samples in both datasets. We then calculated Pearson correlation between gene expression and histone modification measurements across these age-matched autism and control samples.

Functional enrichment analysis

Enrichment analysis of function terms or pathways was performed as described previously (Liu et al., 2012; Somel et al., 2011). Briefly, human Kyoto Encylopedia of Genes and Genomes (KEGG) pathway annotation (Kanehisa et al., 2008) and Gene Ontology (GO) annotation (Ashburner et al., 2000) were downloaded from KEGG (http://www.genome.jp/kegg/) and Ensembl databases, respectively. We used the GO "term" and "graph path" tables downloaded from the GO database (http://archive.geneontology.org/latest-termdb/) to associate each GO term with higher terms. To identify overrepresented KEGG pathways and GO terms, we used the hypergeometric test and adjusted p-values for multiple-testing using Benjamini-Hochberg correction. Only pathways/terms containing more than three genes were tested.

Enrichment analysis of cell type-specific genes was performed as previously described (Somel et al., 2009a). Briefly, we used 1,155 and 578 human genes described to be enriched in gray and white matter from the human frontal cortex (Erraji-Benchekroun et al., 2005). For the cell-type analysis, we used expression levels measured from purified mouse neurons, astrocytes, and oligodendrocytes (Cahoy et al., 2008). We defined 1116, 965, and 991 human-mouse one-to-one orthologs as neuron-, astrocyte-, and oligodendrocyte-specific, using an effect size cutoff of 2. Additionally, we used the marker genes of cell types and cortical layers reported in (Courchesne et al., 2011; Hutsler and Zhang, 2010) to test their enrichment within each cluster.

Supporting References

Ashburner, M., Ball, C., Blake, J., Botstein, D., Butler, H., Cherry, J., Davis, A., Dolinski, K., Dwight, S., Eppig, J., *et al.* (2000). Gene Ontology: tool for the unification of biology. Nat. Genet. *25*, 25-29.

Boedigheimer, M.J., Wolfinger, R.D., Bass, M.B., Bushel, P.R., Chou, J.W., Cooper, M., Corton, J.C., Fostel, J., Hester, S., Lee, J.S., *et al.* (2008). Sources of variation in baseline gene expression levels from toxicogenomics study control animals across multiple laboratories. BMC Genomics *9*, 285.

Cahoy, J.D., Emery, B., Kaushal, A., Foo, L.C., Zamanian, J.L., Christopherson, K.S., Xing, Y., Lubischer, J.L., Krieg, P.A., Krupenko, S.A., *et al.* (2008). A transcriptome database for astrocytes, neurons, and oligodendrocytes: a new resource for understanding brain development and function. J. Neurosci. *28*, 264-278.

Cheung, I., Shulha, H.P., Jiang, Y., Matevossian, A., Wang, J., Weng, Z., and Akbarian, S. (2010). Developmental regulation and individual differences of neuronal H3K4me3 epigenomes in the prefrontal cortex. Proc. Natl. Acad. Sci. U. S. A. *107*, 8824-8829.

Courchesne, E., Mouton, P.R., Calhoun, M.E., Semendeferi, K., Ahrens-Barbeau, C., Hallet, M.J., Barnes, C.C., and Pierce, K. (2011). Neuron number and size in prefrontal cortex of children with autism. JAMA *306*, 2001-2010.

Erraji-Benchekroun, L., Underwood, M., Arango, V., Galfalvy, H., Pavlidis, P., Smyrniotopoulos, P., Mann, J., and Sibille, E. (2005). Molecular aging in human prefrontal cortex is selective and continuous throughout adult life. Biol Psychiatry. *57*, 549.

Faraway, J. (2002). Practical Regression and Anova Using R. Available at: http://cran.rproject.org/doc/contrib/Faraway-PRA.pdf. .

Fu, X., Giavalisco, P., Liu, X., Catchpole, G., Fu, N., Ning, Z.-B., Guo, S., Yan, Z., Somel, M., Pääbo, S., *et al.* (2011). Rapid metabolic evolution in human prefrontal cortex. Proc. Natl. Acad. Sci. U. S. A.

Hutsler, J.J., and Zhang, H. (2010). Increased dendritic spine densities on cortical projection neurons in autism spectrum disorders. Brain Res. *1309*, 83-94.

Kanehisa, M., Araki, M., Goto, S., Hattori, M., Hirakawa, M., Itoh, M., Katayama, T., Kawashima, S., Okuda, S., Tokimatsu, T., *et al.* (2008). KEGG for linking genomes to life and the environment. Nucleic Acids Res. *36*, D480-484.

Langmead, B., Trapnell, C., Pop, M., and Salzberg, S.L. (2009). Ultrafast and memory-efficient alignment of short DNA sequences to the human genome. Genome Biol *10*, R25.

Li, J., Bushel, P., Chu, T.-M., and Wolfinger, R. (2009). Principal Variance Components Analysis: Estimating Batch Effects in Microarray Gene Expression Data. In Batch Effects and

Noise in Microarray Experiments: Sources and Solutions, S. A, ed. (Chichester: John Wiley & Sons, Ltd), pp. 141-154.
Liu, X., Somel, M., Tang, L., Yan, Z., Jiang, X., Guo, S., Yuan, Y., He, L., Oleksiak, A., Zhang, Y., *et al.* (2012). Extension of cortical synaptic development distinguishes humans from chimpanzees and macaques. Genome Res. *22*, 611-622.
Shulha, H.P., Cheung, I., Whittle, C., Wang, J., Virgil, D., Lin, C.L., Guo, Y., Lessard, A., Akbarian, S., and Weng, Z. (2011). Epigenetic signatures of autism: trimethylated H3K4 landscapes in prefrontal neurons. Arch. Gen. Psychiatry *69*, 314-324.
Somel, M., Franz, H., Yan, Z., Lorenc, A., Guo, S., Giger, T., Kelso, J., Nickel, B., Dannemann, M., Bahn, S., *et al.* (2009a). Transcriptional neoteny in the human brain. Proc Natl Acad Sci USA *106*, 5743-5748.
Somel, M., Franz, H., Yan, Z., Lorenc, A., Guo, S., Giger, T., Kelso, J., Nickel, B., Dannemann, M., Bahn, S., *et al.* (2009b). Transcriptional neoteny in the human brain. Proc. Natl. Acad. Sci. U. S. A. *106*, 5743-5748.
Somel, M., Liu, X., Tang, L., Yan, Z., Hu, H., Guo, S., Jiang, X., Zhang, X., Xu, G., Xie, G., *et al.* (2011). MicroRNA-Driven Developmental Remodeling in the Brain Distinguishes Humans from Other Primates. PLoS Biol. *9*, e1001214.
Voineagu, I., Wang, X., Johnston, P., Lowe, J.K., Tian, Y., Horvath, S., Mill, J., Cantor, R.M., Blencowe, B.J., and Geschwind, D.H. (2011). Transcriptomic analysis of autistic brain reveals convergent molecular pathology. Nature *474*, 380-384.
Wang, L., Wang, S., and Li, W. (2012). RSeQC: quality control of RNA-seq experiments. Bioinformatics *28*, 2184-2185.
